# Supplementary material for: cDNA Transcriptome of Arabidopsis Reveals Various Defense Priming Induced by a Broad-Spectrum Biocontrol Agent Burkholderia sp. SSG
Source: Int J Mol Sci. 2022 Mar 15;23(6):3151. doi: 10.3390/ijms23063151 (PMC8954528; doi:10.3390/ijms23063151)
Supplement: Supplementary file 1 [file ijms-23-03151-s001.zip › Sumplementary/Fig. S1.pdf]

**6 hpt**

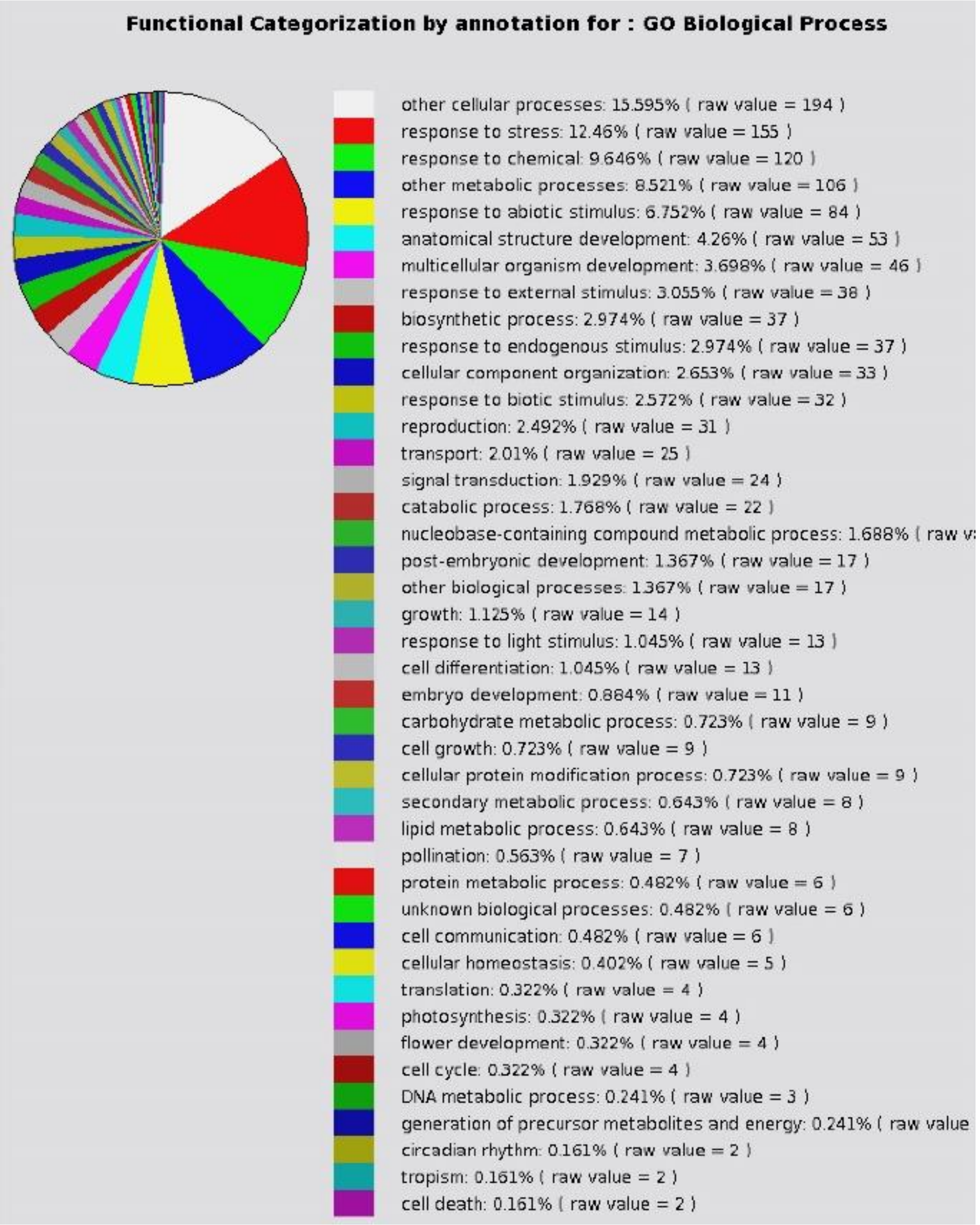
Functional Categorization by annotation for : GO Biological Process
other cellular processes: 15.595% ( raw value = 194 )
response to stress: 12.46% ( raw value = 155 )
response to chemical: 9.646% ( raw value = 120 )
other metabolic processes: 8.521% ( raw value = 106 )
response to abiotic stimulus: 6.752% ( raw value = 84 )
anatomical structure development: 4.26% ( raw value = 53 )
multicellular organism development: 3.698% ( raw value = 46 )
response to external stimulus: 3.055% ( raw value = 38 )
biosynthetic process: 2.974% ( raw value = 37 )
response to endogenous stimulus: 2.974% ( raw value = 37 )
cellular component organization: 2.653% ( raw value = 33 )
response to biotic stimulus: 2.572% ( raw value = 32 )
reproduction: 2.492% ( raw value = 31 )
transport: 2.01% ( raw value = 25 )
signal transduction: 1.929% ( raw value = 24 )
catabolic process: 1.768% ( raw value = 22 )
nucleobase-containing compound metabolic process: 1.688% ( raw v
post-embryonic development: 1.367% ( raw value = 17 )
other biological processes: 1.367% ( raw value = 17 )
growth: 1.125% ( raw value = 14 )
response to light stimulus: 1.045% ( raw value = 13 )
cell differentiation: 1.045% ( raw value = 13 )
embryo development: 0.884% ( raw value = 11 )
carbohydrate metabolic process: 0.723% ( raw value = 9 )
cell growth: 0.723% ( raw value = 9 )
cellular protein modification process: 0.723% ( raw value = 9 )
secondary metabolic process: 0.643% ( raw value = 8 )
lipid metabolic process: 0.643% ( raw value = 8 )
pollination: 0.563% ( raw value = 7 )
protein metabolic process: 0.482% ( raw value = 6 )
unknown biological processes: 0.482% ( raw value = 6 )
cell communication: 0.482% ( raw value = 6 )
cellular homeostasis: 0.402% ( raw value = 5 )
translation: 0.322% ( raw value = 4 )
photosynthesis: 0.322% ( raw value = 4 )
flower development: 0.322% ( raw value = 4 )
cell cycle: 0.322% ( raw value = 4 )
DNA metabolic process: 0.241% ( raw value = 3 )
generation of precursor metabolites and energy: 0.241% ( raw value
circadian rhythm: 0.161% ( raw value = 2 )
tropism: 0.161% ( raw value = 2 )
cell death: 0.161% ( raw value = 2 )

**24 hpt**

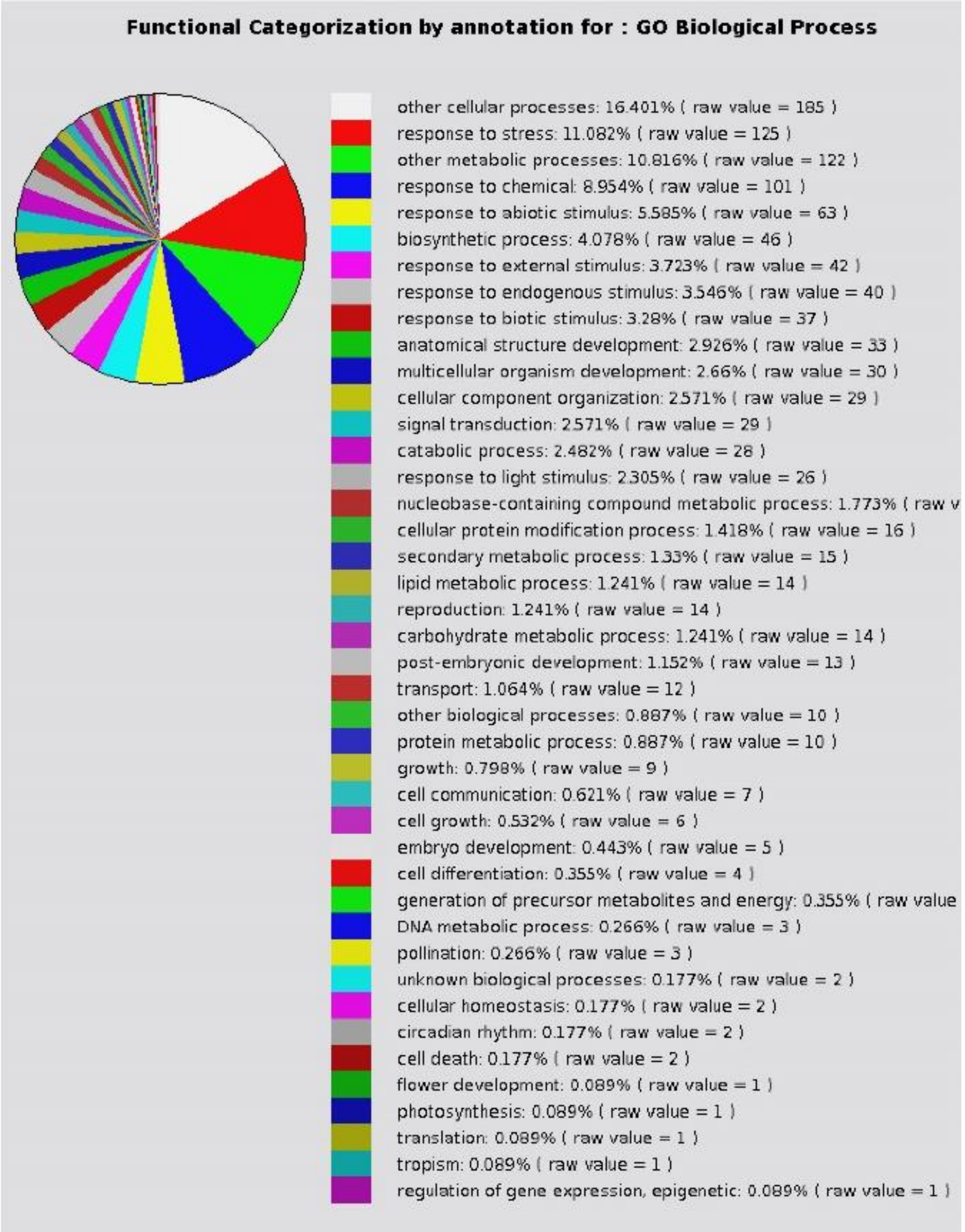
Functional Categorization by annotation for : GO Biological Process
other cellular processes: 16.401% ( raw value = 185 )
response to stress: 11.082% ( raw value = 125 )
other metabolic processes: 10.816% ( raw value = 122 )
response to chemical: 8.954% ( raw value = 101 )
response to abiotic stimulus: 5.585% ( raw value = 63 )
biosynthetic process: 4.078% ( raw value = 46 )
response to external stimulus: 3.723% ( raw value = 42 )
response to endogenous stimulus: 3.546% ( raw value = 40 )
response to biotic stimulus: 3.28% ( raw value = 37 )
anatomical structure development: 2.926% ( raw value = 33 )
multicellular organism development: 2.66% ( raw value = 30 )
cellular component organization: 2.571% ( raw value = 29 )
signal transduction: 2.571% ( raw value = 29 )
catabolic process: 2.482% ( raw value = 28 )
response to light stimulus: 2.305% ( raw value = 26 )
nucleobase-containing compound metabolic process: 1.773% ( raw v
cellular protein modification process: 1.418% ( raw value = 16 )
secondary metabolic process: 1.33% ( raw value = 15 )
lipid metabolic process: 1.241% ( raw value = 14 )
reproduction: 1.241% ( raw value = 14 )
carbohydrate metabolic process: 1.241% ( raw value = 14 )
post-embryonic development: 1.152% ( raw value = 13 )
transport: 1.064% ( raw value = 12 )
other biological processes: 0.887% ( raw value = 10 )
protein metabolic process: 0.887% ( raw value = 10 )
growth: 0.798% ( raw value = 9 )
cell communication: 0.621% ( raw value = 7 )
cell growth: 0.532% ( raw value = 6 )
embryo development: 0.443% ( raw value = 5 )
cell differentiation: 0.355% ( raw value = 4 )
generation of precursor metabolites and energy: 0.355% ( raw value
DNA metabolic process: 0.266% ( raw value = 3 )
pollination: 0.266% ( raw value = 3 )
unknown biological processes: 0.177% ( raw value = 2 )
cellular homeostasis: 0.177% ( raw value = 2 )
circadian rhythm: 0.177% ( raw value = 2 )
cell death: 0.177% ( raw value = 2 )
flower development: 0.089% ( raw value = 1 )
photosynthesis: 0.089% ( raw value = 1 )
translation: 0.089% ( raw value = 1 )
tropism: 0.089% ( raw value = 1 )
regulation of gene expression, epigenetic: 0.089% ( raw value = 1 )
