# Supplementary material for: cDNA Transcriptome of Arabidopsis Reveals Various Defense Priming Induced by a Broad-Spectrum Biocontrol Agent Burkholderia sp. SSG
Source: Int J Mol Sci. 2022 Mar 15;23(6):3151. doi: 10.3390/ijms23063151 (PMC8954528; doi:10.3390/ijms23063151)
Supplement: Supplementary file 1 [file ijms-23-03151-s001.zip › Sumplementary/Fig. S2.pdf]

6 hpt

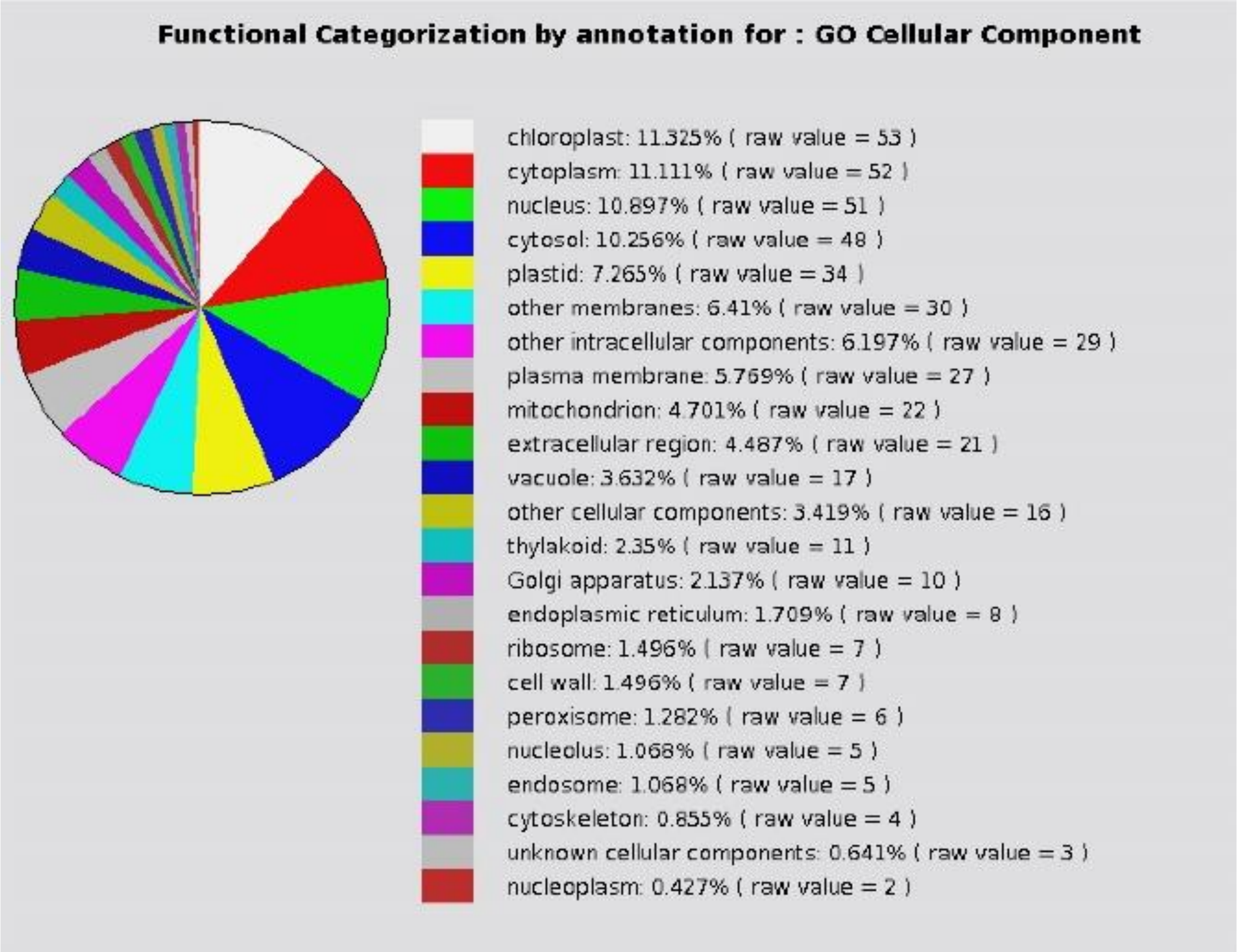
Functional Categorization by annotation for : GO Cellular Component
chloroplast: 11.325% (raw value = 53)
cytoplasm: 11.111% (raw value = 52)
nucleus: 10.897% (raw value = 51)
cytosol: 10.256% (raw value = 48)
plastid: 7.265% (raw value = 34)
other membranes: 6.41% (raw value = 30)
other intracellular components: 6.197% (raw value = 29)
plasma membrane: 5.769% (raw value = 27)
mitochondrion: 4.701% (raw value = 22)
extracellular region: 4.487% (raw value = 21)
vacuole: 3.632% (raw value = 17)
other cellular components: 3.419% (raw value = 16)
thylakoid: 2.35% (raw value = 11)
Golgi apparatus: 2.137% (raw value = 10)
endoplasmic reticulum: 1.709% (raw value = 8)
ribosome: 1.496% (raw value = 7)
cell wall: 1.496% (raw value = 7)
peroxisome: 1.282% (raw value = 6)
nucleolus: 1.068% (raw value = 5)
endosome: 1.068% (raw value = 5)
cytoskeleton: 0.855% (raw value = 4)
unknown cellular components: 0.641% (raw value = 3)
nucleoplasm: 0.427% (raw value = 2)

24 hpt

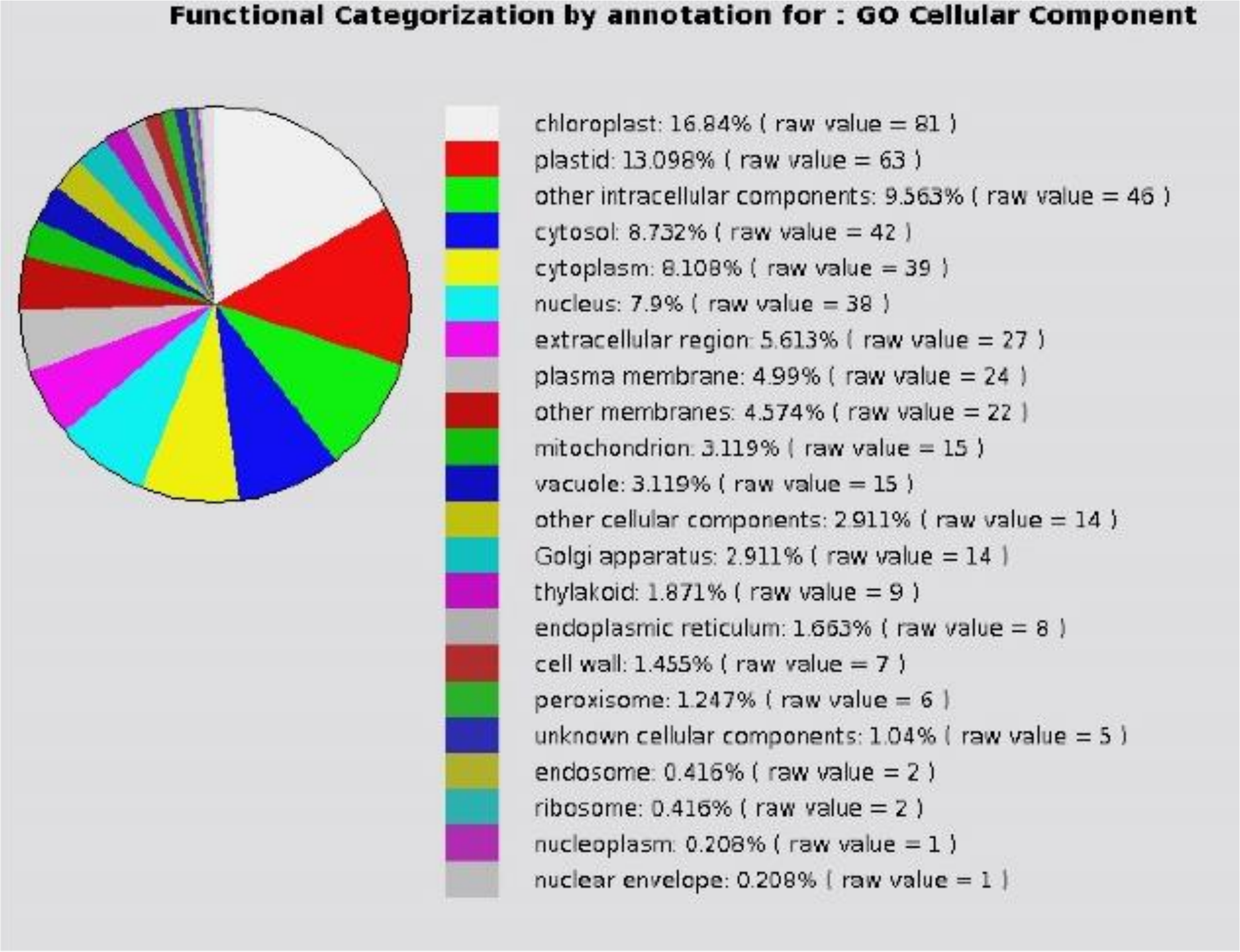
Functional Categorization by annotation for : GO Cellular Component
chloroplast: 16.84% (raw value = 81)
plastid: 13.098% (raw value = 63)
other intracellular components: 9.563% (raw value = 46)
cytosol: 8.732% (raw value = 42)
cytoplasm: 8.108% (raw value = 39)
nucleus: 7.9% (raw value = 38)
extracellular region: 5.613% (raw value = 27)
plasma membrane: 4.99% (raw value = 24)
other membranes: 4.574% (raw value = 22)
mitochondrion: 3.119% (raw value = 15)
vacuole: 3.119% (raw value = 15)
other cellular components: 2.911% (raw value = 14)
Golgi apparatus: 2.911% (raw value = 14)
thylakoid: 1.871% (raw value = 9)
endoplasmic reticulum: 1.663% (raw value = 8)
cell wall: 1.455% (raw value = 7)
peroxisome: 1.247% (raw value = 6)
unknown cellular components: 1.04% (raw value = 5)
endosome: 0.416% (raw value = 2)
ribosome: 0.416% (raw value = 2)
nucleoplasm: 0.208% (raw value = 1)
nuclear envelope: 0.208% (raw value = 1)
