# Supplementary material for: cDNA Transcriptome of Arabidopsis Reveals Various Defense Priming Induced by a Broad-Spectrum Biocontrol Agent Burkholderia sp. SSG
Source: Int J Mol Sci. 2022 Mar 15;23(6):3151. doi: 10.3390/ijms23063151 (PMC8954528; doi:10.3390/ijms23063151)
Supplement: Supplementary file 1 [file ijms-23-03151-s001.zip › Sumplementary/Fig. S3.pdf]

6 hpt

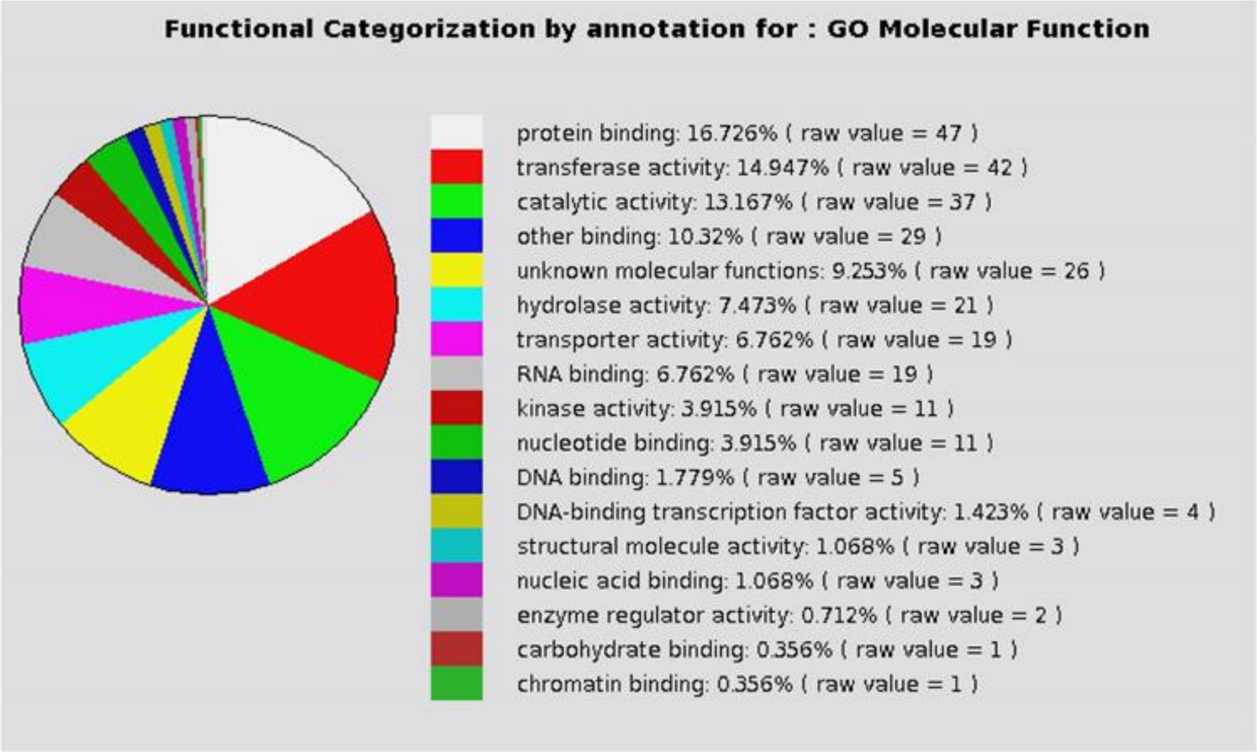
Functional Categorization by annotation for : GO Molecular Function
protein binding: 16.726% (raw value = 47)
transferase activity: 14.947% (raw value = 42)
catalytic activity: 13.167% (raw value = 37)
other binding: 10.32% (raw value = 29)
unknown molecular functions: 9.253% (raw value = 26)
hydrolase activity: 7.473% (raw value = 21)
transporter activity: 6.762% (raw value = 19)
RNA binding: 6.762% (raw value = 19)
kinase activity: 3.915% (raw value = 11)
nucleotide binding: 3.915% (raw value = 11)
DNA binding: 1.779% (raw value = 5)
DNA-binding transcription factor activity: 1.423% (raw value = 4)
structural molecule activity: 1.068% (raw value = 3)
nucleic acid binding: 1.068% (raw value = 3)
enzyme regulator activity: 0.712% (raw value = 2)
carbohydrate binding: 0.356% (raw value = 1)
chromatin binding: 0.356% (raw value = 1)

24 hpt

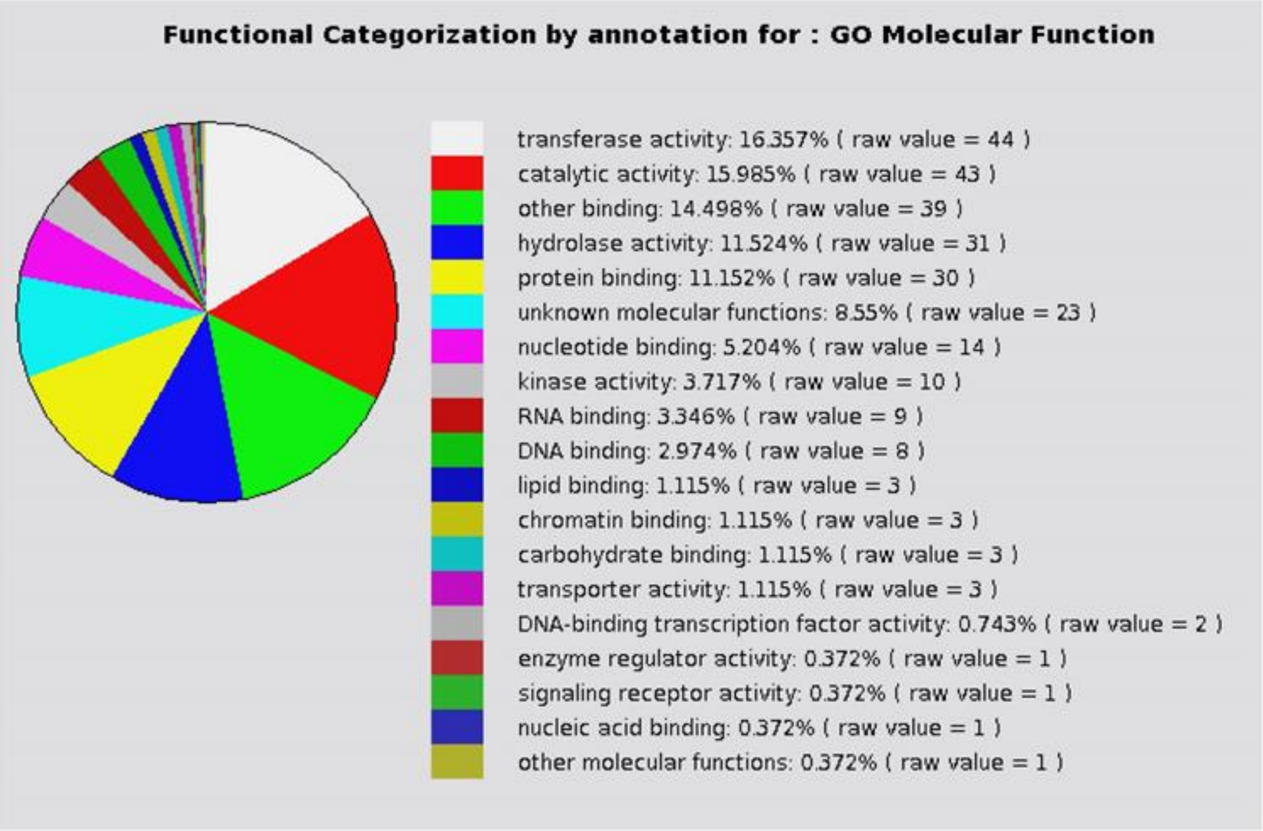
Functional Categorization by annotation for : GO Molecular Function
transferase activity: 16.357% (raw value = 44)
catalytic activity: 15.985% (raw value = 43)
other binding: 14.498% (raw value = 39)
hydrolase activity: 11.524% (raw value = 31)
protein binding: 11.152% (raw value = 30)
unknown molecular functions: 8.55% (raw value = 23)
nucleotide binding: 5.204% (raw value = 14)
kinase activity: 3.717% (raw value = 10)
RNA binding: 3.346% (raw value = 9)
DNA binding: 2.974% (raw value = 8)
lipid binding: 1.115% (raw value = 3)
chromatin binding: 1.115% (raw value = 3)
carbohydrate binding: 1.115% (raw value = 3)
transporter activity: 1.115% (raw value = 3)
DNA-binding transcription factor activity: 0.743% (raw value = 2)
enzyme regulator activity: 0.372% (raw value = 1)
signaling receptor activity: 0.372% (raw value = 1)
nucleic acid binding: 0.372% (raw value = 1)
other molecular functions: 0.372% (raw value = 1)
