# Supplementary material for: cDNA Transcriptome of Arabidopsis Reveals Various Defense Priming Induced by a Broad-Spectrum Biocontrol Agent Burkholderia sp. SSG
Source: Int J Mol Sci. 2022 Mar 15;23(6):3151. doi: 10.3390/ijms23063151 (PMC8954528; doi:10.3390/ijms23063151)
Supplement: Supplementary file 1 [file ijms-23-03151-s001.zip › Sumplementary/Fig. S4.pdf]

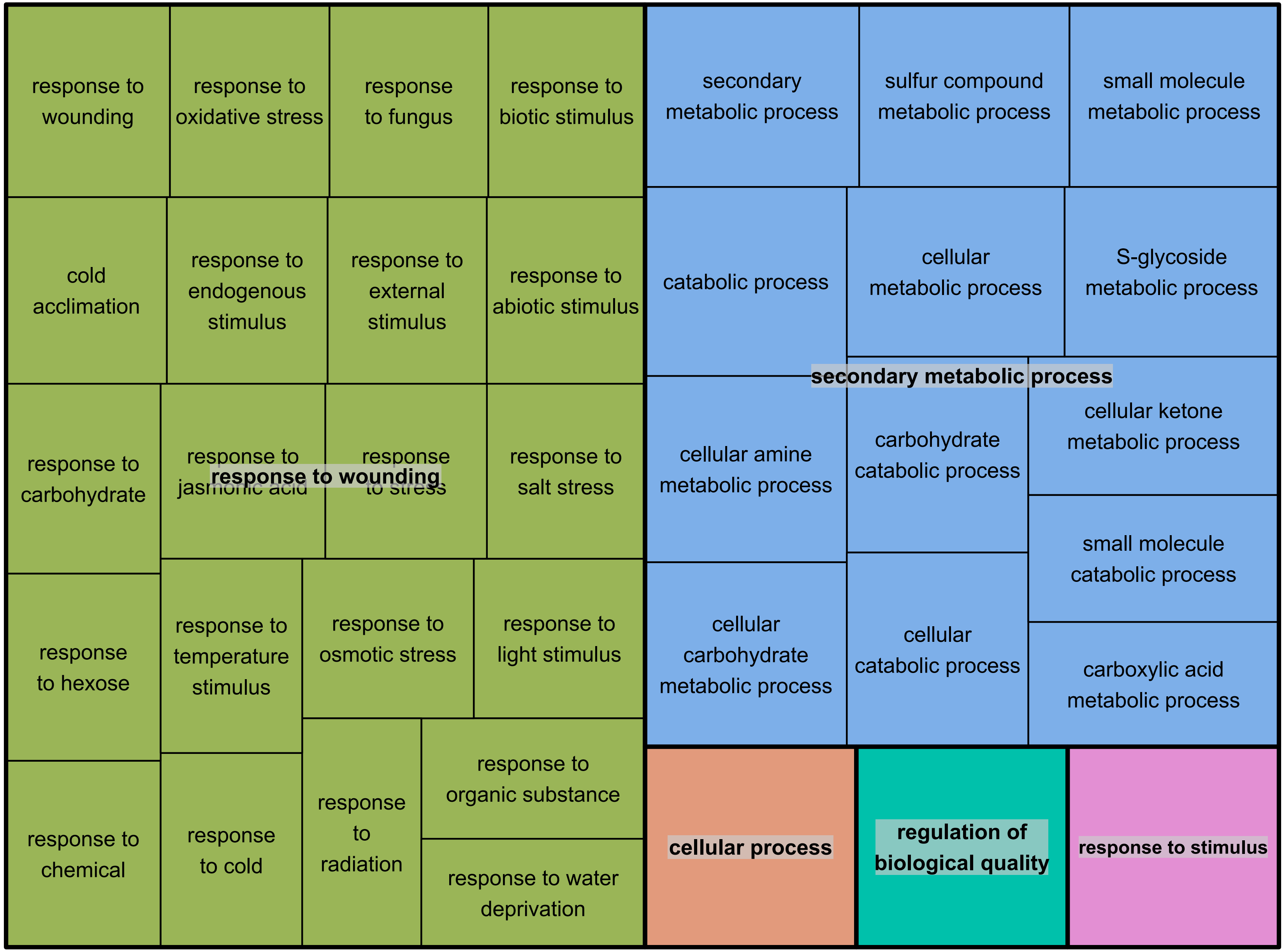
response to wounding
response to oxidative stress
response to fungus
response to biotic stimulus
cold acclimation
response to endogenous stimulus
response to external stimulus
response to abiotic stimulus
response to carbohydrate
response to jasmonic acid
response to stress
response to wounding
response to salt stress
response to hexose
response to temperature stimulus
response to osmotic stress
response to light stimulus
response to chemical
response to cold
response to radiation
response to organic substance
response to water deprivation
secondary metabolic process
sulfur compound metabolic process
small molecule metabolic process
catabolic process
cellular metabolic process
S-glycoside metabolic process
secondary metabolic process
cellular amine metabolic process
carbohydrate catabolic process
cellular ketone metabolic process
small molecule catabolic process
cellular carbohydrate metabolic process
cellular catabolic process
carboxylic acid metabolic process
cellular process
regulation of biological quality
response to stimulus
