# Supplementary material for: cDNA Transcriptome of Arabidopsis Reveals Various Defense Priming Induced by a Broad-Spectrum Biocontrol Agent Burkholderia sp. SSG
Source: Int J Mol Sci. 2022 Mar 15;23(6):3151. doi: 10.3390/ijms23063151 (PMC8954528; doi:10.3390/ijms23063151)
Supplement: Supplementary file 1 [file ijms-23-03151-s001.zip › Sumplementary/Fig. S5.pdf]

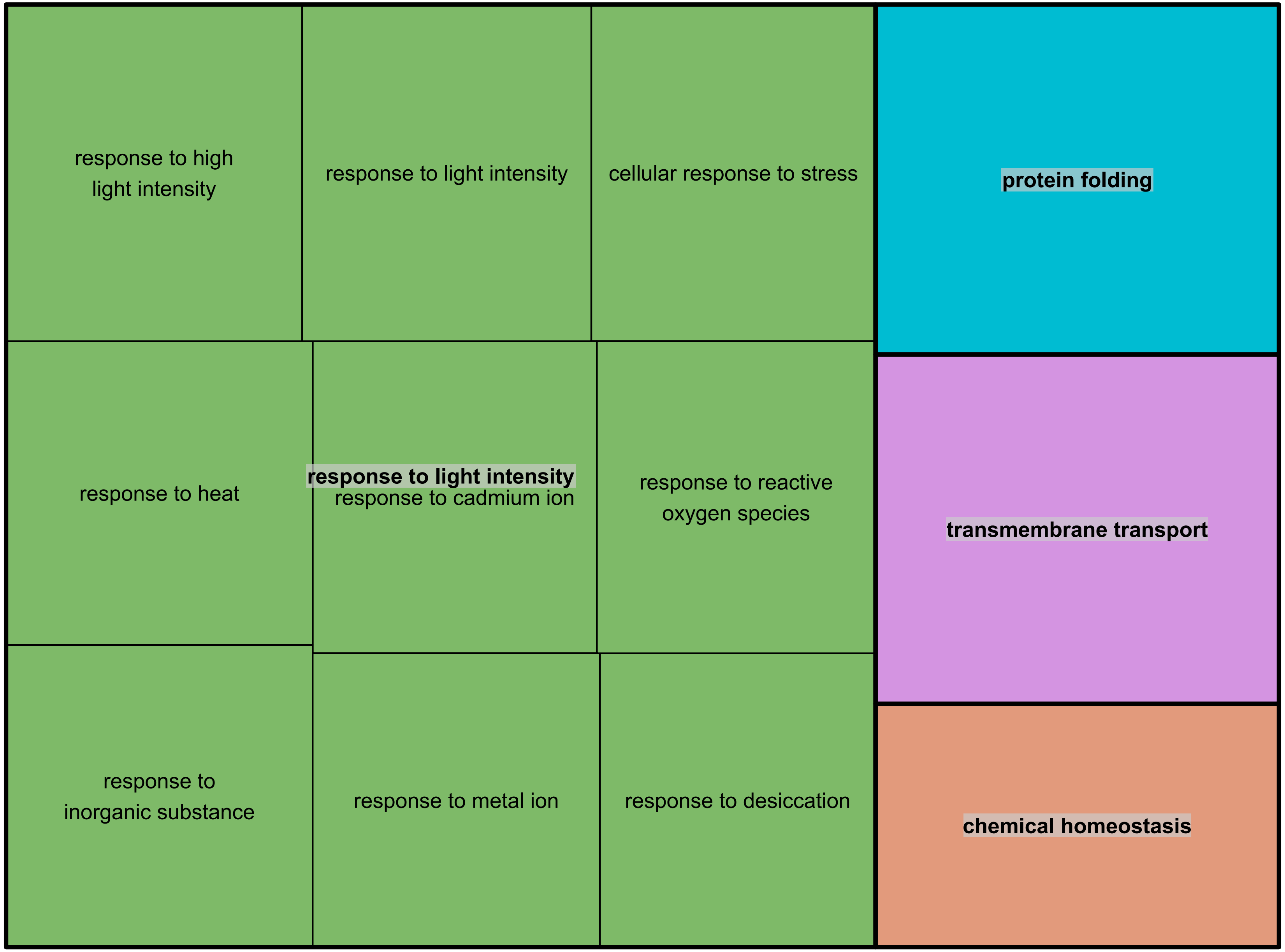
response to high light intensity
response to light intensity
cellular response to stress
protein folding
response to heat
response to light intensity
response to cadmium ion
response to reactive oxygen species
transmembrane transport
response to inorganic substance
response to metal ion
response to desiccation
chemical homeostasis
