# Supplementary material for: cDNA Transcriptome of Arabidopsis Reveals Various Defense Priming Induced by a Broad-Spectrum Biocontrol Agent Burkholderia sp. SSG
Source: Int J Mol Sci. 2022 Mar 15;23(6):3151. doi: 10.3390/ijms23063151 (PMC8954528; doi:10.3390/ijms23063151)
Supplement: Supplementary file 1 [file ijms-23-03151-s001.zip › Sumplementary/Fig. S6.pdf]

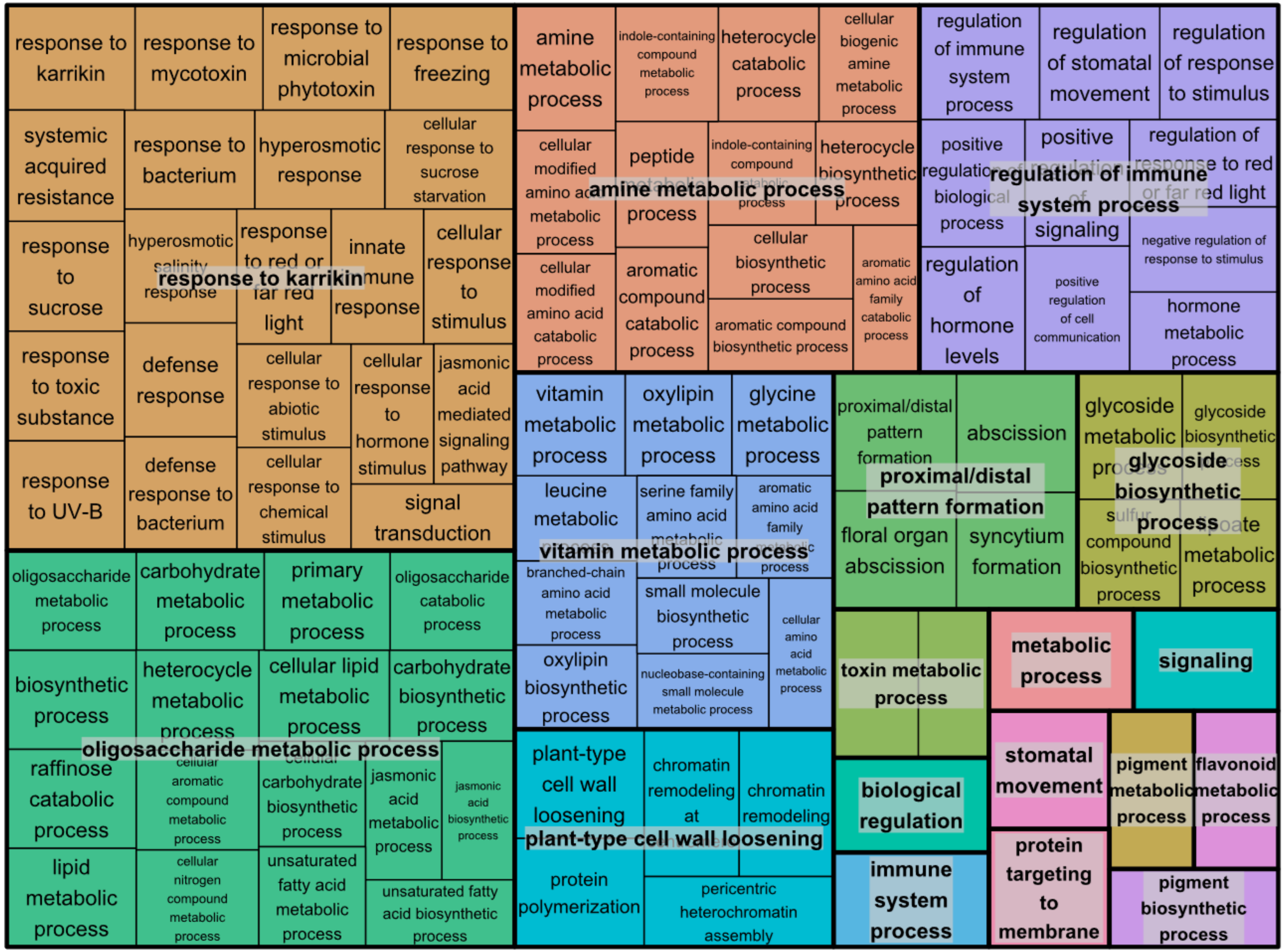
response to karrikin
response to mycotoxin
response to microbial phytotoxin
response to freezing
systemic acquired resistance
response to bacterium
hyperosmotic response
cellular response to sucrose starvation
response to sucrose
hyperosmotic salinity response
response to red or far red light
innate immune response
cellular response to stimulus
response to karrikin
response to toxic substance
defense response
cellular response to abiotic stimulus
cellular response to hormone stimulus
jasmonic acid mediated signaling pathway
response to UV-B
defense response to bacterium
cellular response to chemical stimulus
signal transduction
amine metabolic process
indole-containing compound metabolic process
heterocycle catabolic process
cellular biogenic amine metabolic process
cellular modified amino acid metabolic process
peptide metabolic process
indole-containing compound catabolic process
heterocycle biosynthetic process
amine metabolic process
cellular biosynthetic process
cellular modified amino acid catabolic process
aromatic compound catabolic process
aromatic compound biosynthetic process
aromatic amino acid family catabolic process
regulation of immune system process
regulation of stomatal movement
regulation of response to stimulus
positive regulation of biological process
positive regulation of signaling
regulation of response to red or far red light
regulation of immune system process
negative regulation of response to stimulus
regulation of hormone levels
positive regulation of cell communication
hormone metabolic process
vitamin metabolic process
oxylipin metabolic process
glycine metabolic process
leucine metabolic process
serine family amino acid metabolic process
aromatic amino acid family metabolic process
vitamin metabolic process
branched-chain amino acid metabolic process
small molecule biosynthetic process
cellular amino acid metabolic process
oxylipin biosynthetic process
nucleobase-containing small molecule metabolic process
proximal/distal pattern formation
abscission
proximal/distal pattern formation
floral organ abscission
syncytium formation
glycoside metabolic process
glycoside biosynthetic process
glycoside biosynthetic process
sulfur compound biosynthetic process
metabolic process
oligosaccharide metabolic process
carbohydrate metabolic process
primary metabolic process
oligosaccharide catabolic process
biosynthetic process
heterocycle metabolic process
cellular lipid metabolic process
carbohydrate biosynthetic process
oligosaccharide metabolic process
raffinose catabolic process
cellular aromatic compound metabolic process
cellular carbohydrate biosynthetic process
jasmonic acid metabolic process
jasmonic acid biosynthetic process
lipid metabolic process
cellular nitrogen compound metabolic process
unsaturated fatty acid metabolic process
unsaturated fatty acid biosynthetic process
plant-type cell wall loosening
chromatin remodeling at
chromatin remodeling
plant-type cell wall loosening
protein polymerization
pericentric heterochromatin assembly
toxin metabolic process
metabolic process
signaling
biological regulation
stomatal movement
pigment metabolic process
flavonoid metabolic process
immune system process
protein targeting to membrane
pigment biosynthetic process
