# Supplementary material for: cDNA Transcriptome of Arabidopsis Reveals Various Defense Priming Induced by a Broad-Spectrum Biocontrol Agent Burkholderia sp. SSG
Source: Int J Mol Sci. 2022 Mar 15;23(6):3151. doi: 10.3390/ijms23063151 (PMC8954528; doi:10.3390/ijms23063151)
Supplement: Supplementary file 1 [file ijms-23-03151-s001.zip › Sumplementary/Fig. S8.pdf]

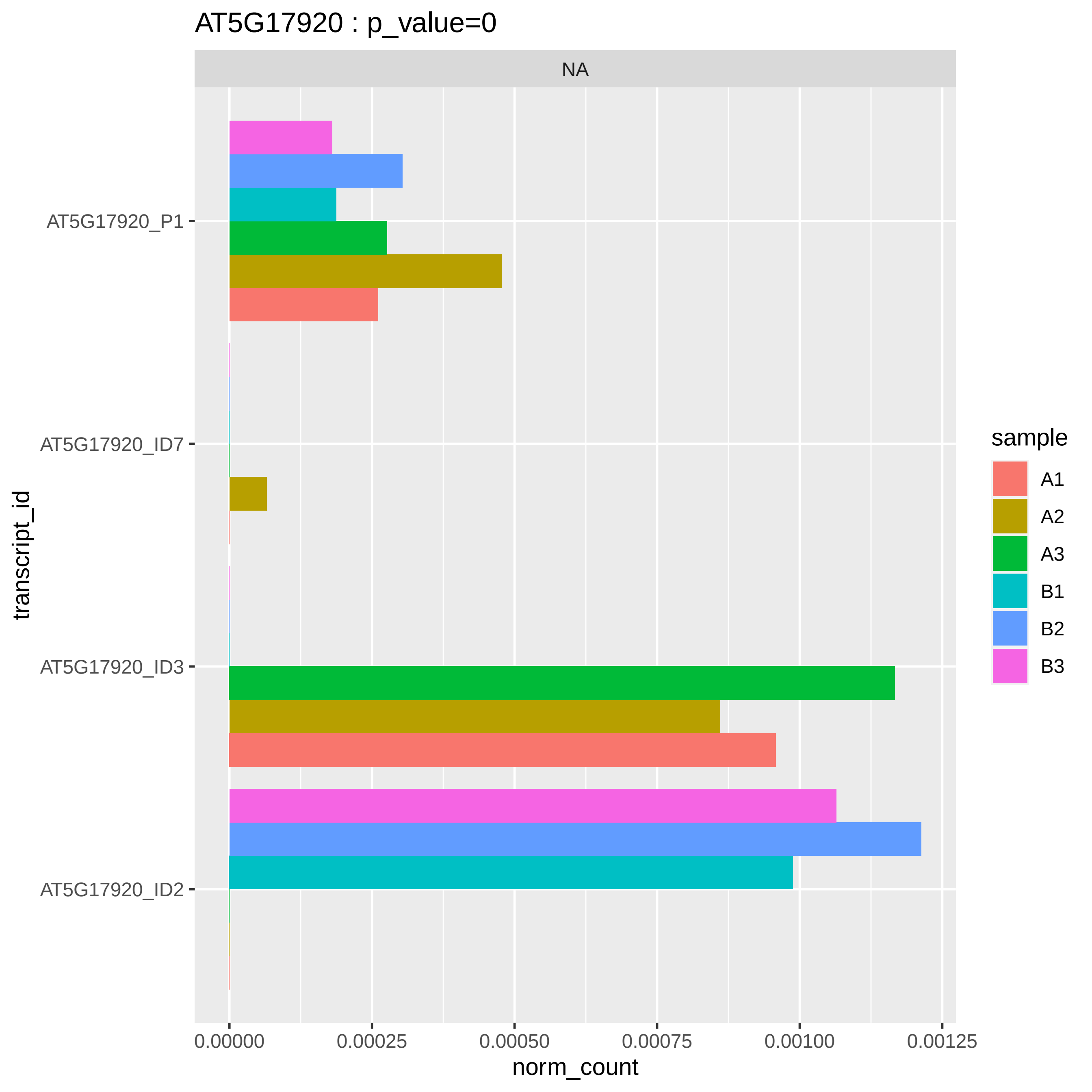
AT5G17920 : p_value=0
NA
AT5G17920_P1
AT5G17920_ID7
AT5G17920_ID3
AT5G17920_ID2
transcript_id
0.00000
0.00025
0.00050
0.00075
0.00100
0.00125
norm_count
sample
A1
A2
A3
B1
B2
B3

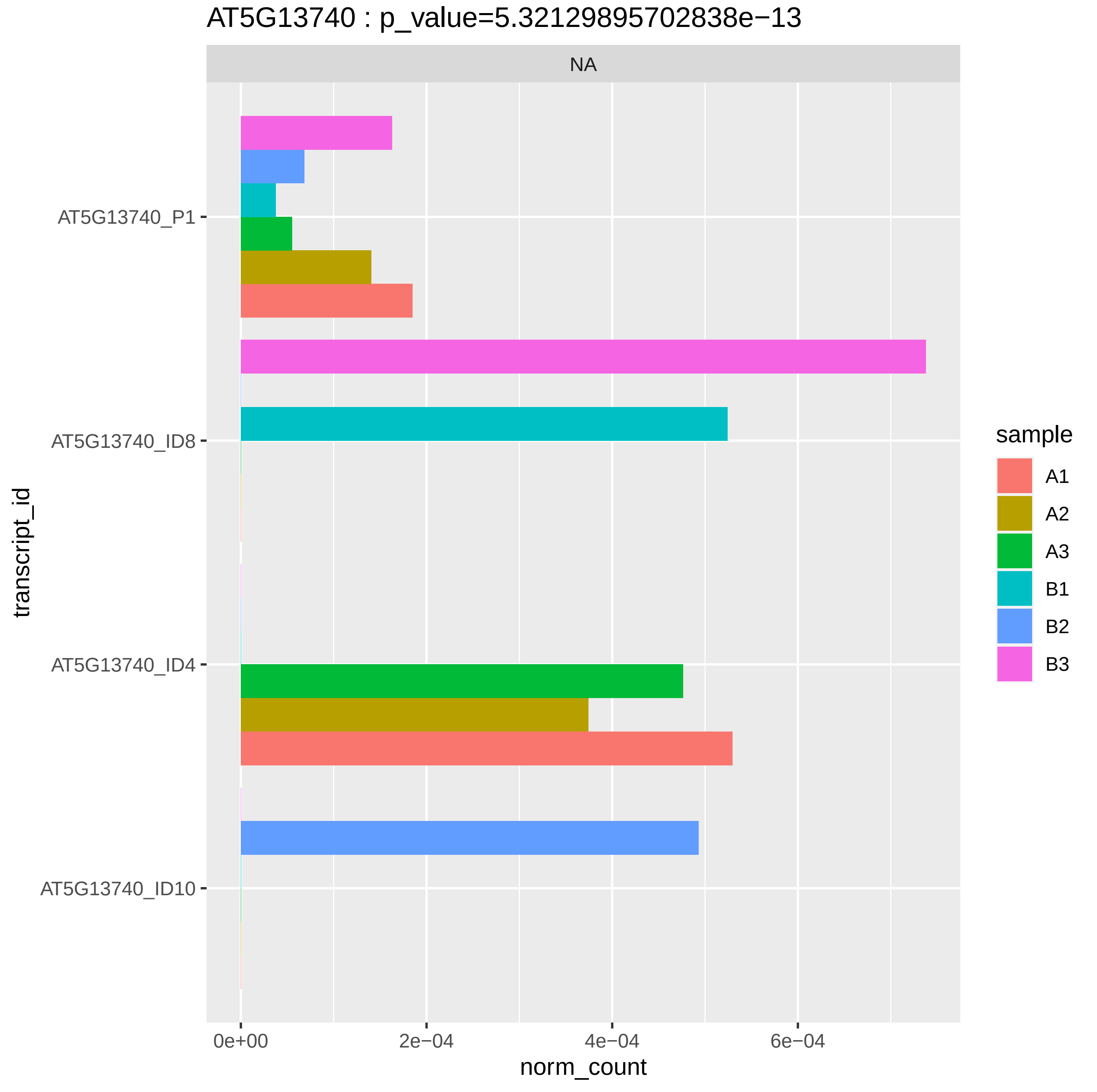

AT5G13740 : p_value=5.32129895702838e−13
NA
AT5G13740_P1
AT5G13740_ID8
AT5G13740_ID4
AT5G13740_ID10
transcript_id
0e+00
2e−04
4e−04
6e−04
norm_count
sample
A1
A2
A3
B1
B2
B3

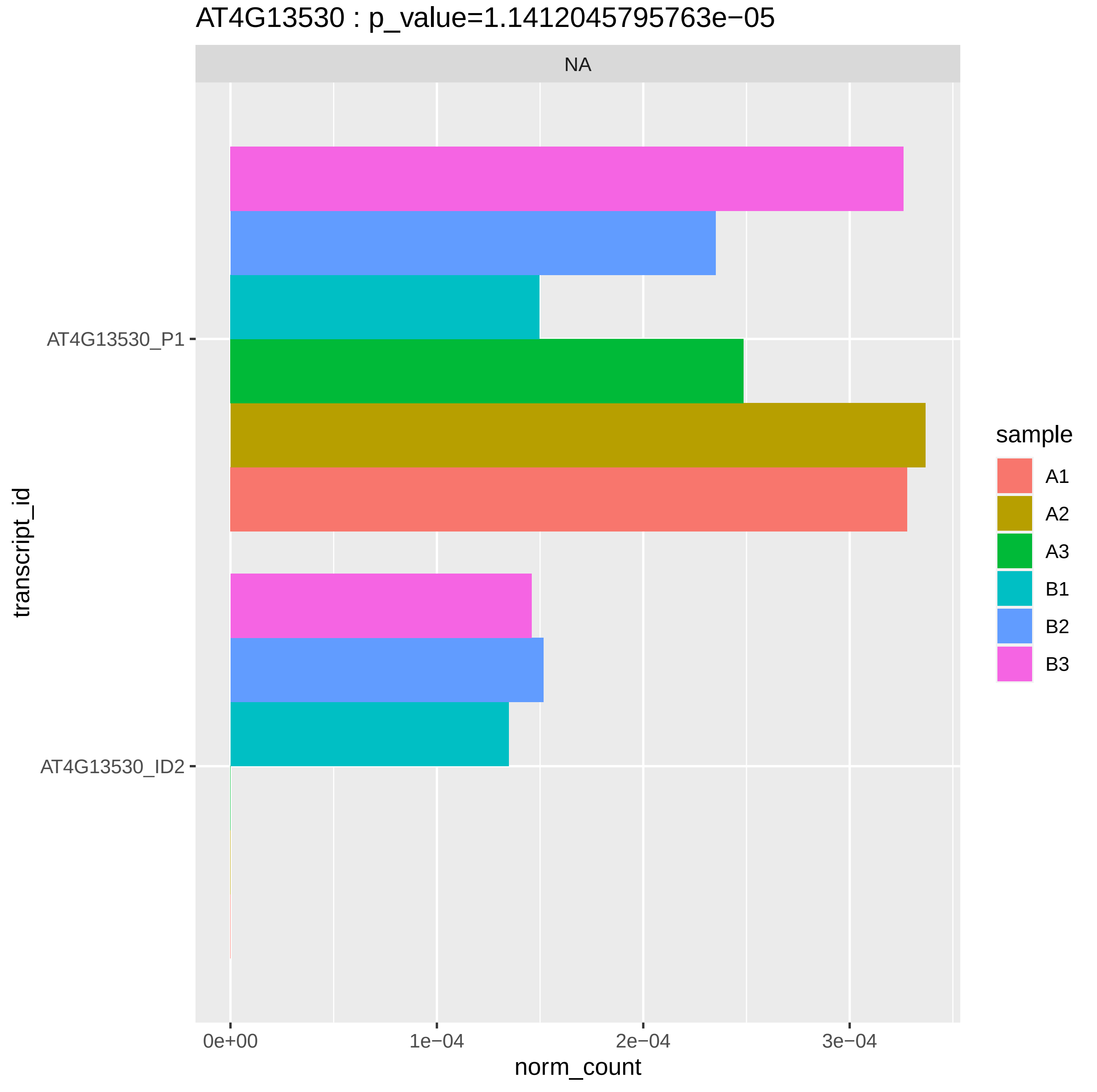
AT4G13530 : p_value=1.1412045795763e−05
NA
AT4G13530_P1
AT4G13530_ID2
transcript_id
0e+00
1e−04
2e−04
3e−04
norm_count
sample
A1
A2
A3
B1
B2
B3

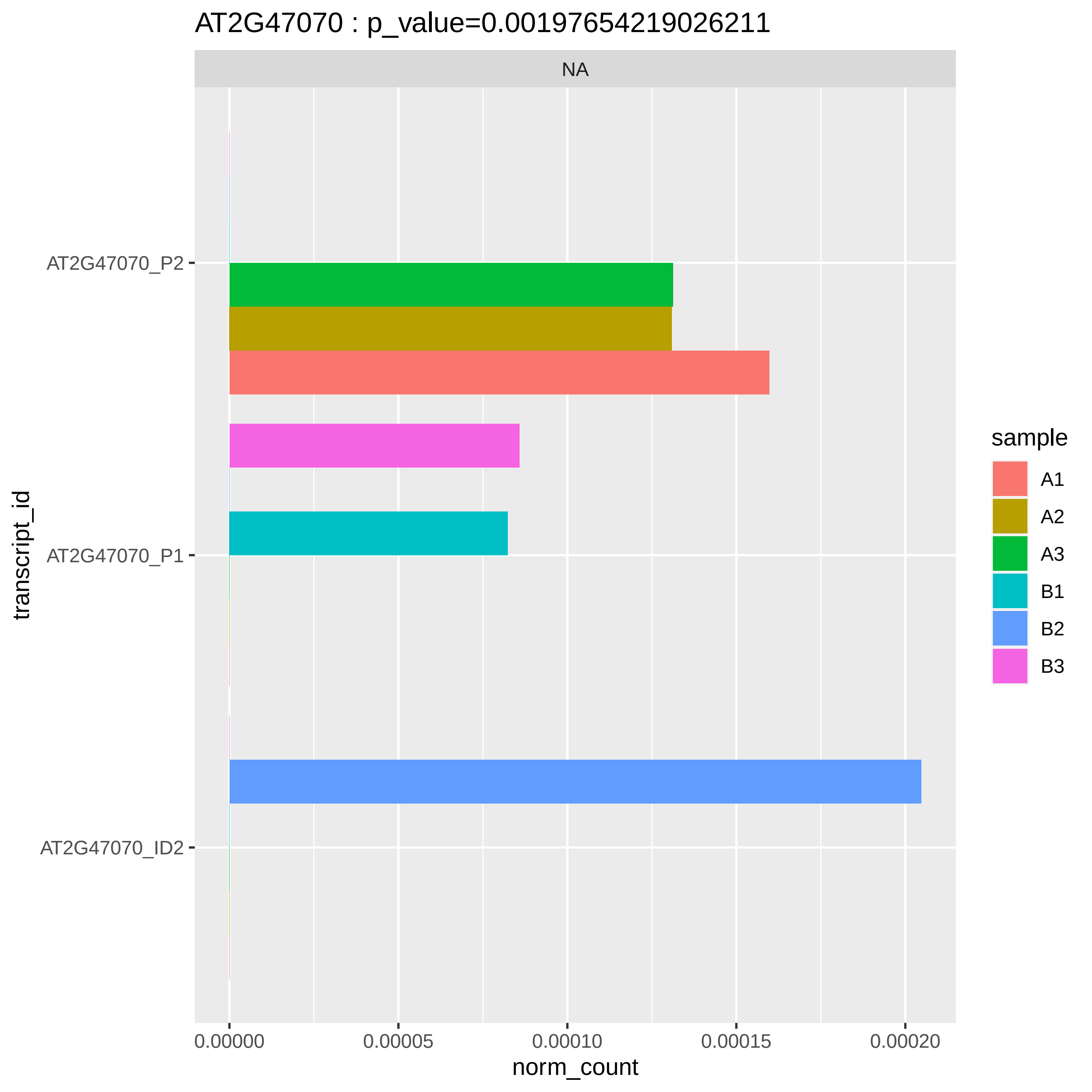

AT2G47070 : p_value=0.00197654219026211
NA
AT2G47070_P2
AT2G47070_P1
AT2G47070_ID2
transcript_id
0.00000
0.00005
0.00010
0.00015
0.00020
norm_count
sample
A1
A2
A3
B1
B2
B3

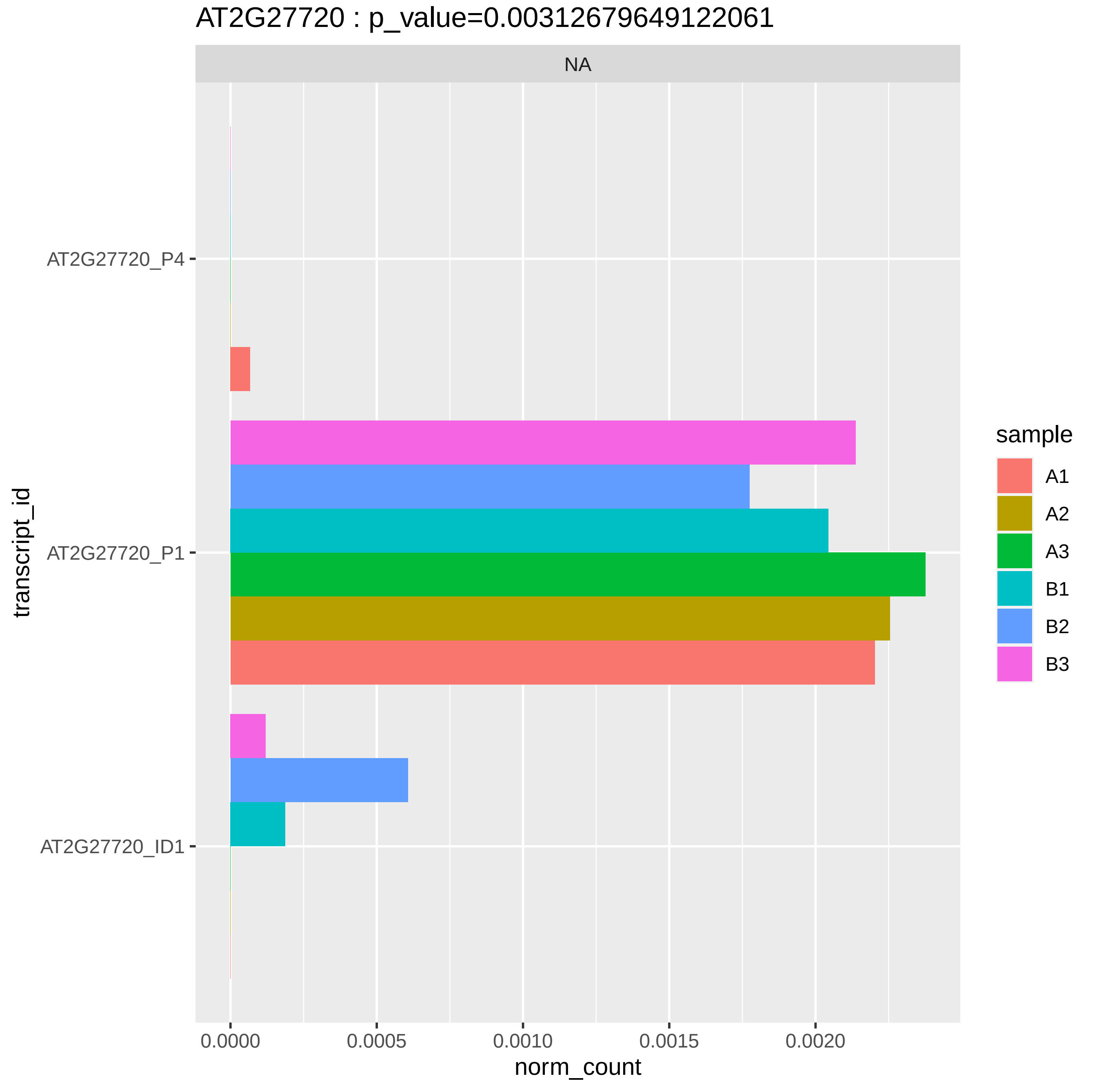
AT2G27720 : p_value=0.00312679649122061
NA
transcript_id
AT2G27720_P4
AT2G27720_P1
AT2G27720_ID1
0.0000
0.0005
0.0010
0.0015
0.0020
norm_count
sample
A1
A2
A3
B1
B2
B3

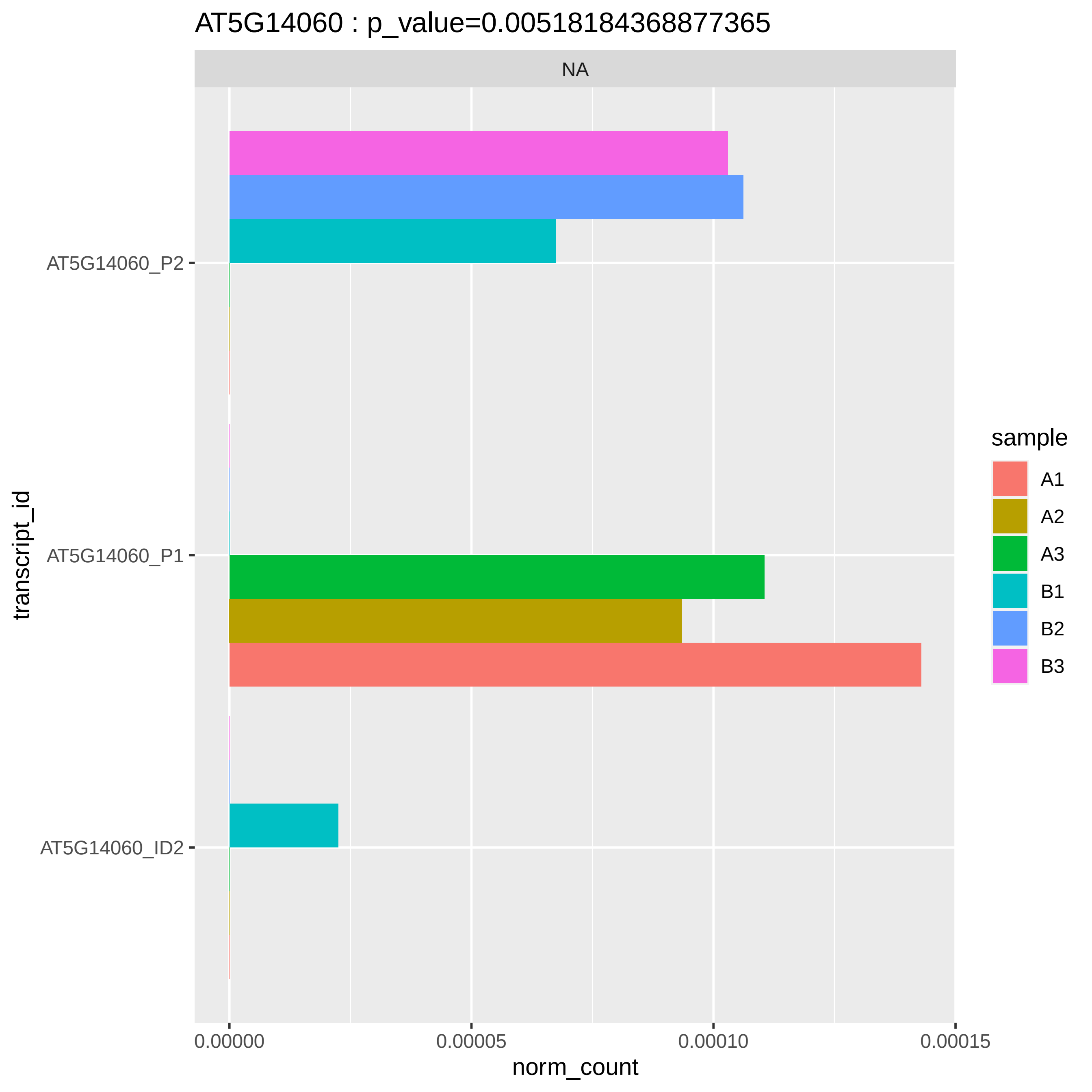
AT5G14060 : p_value=0.00518184368877365
NA
AT5G14060_P2
AT5G14060_P1
AT5G14060_ID2
transcript_id
0.00000
0.00005
0.00010
0.00015
norm_count
sample
A1
A2
A3
B1
B2
B3

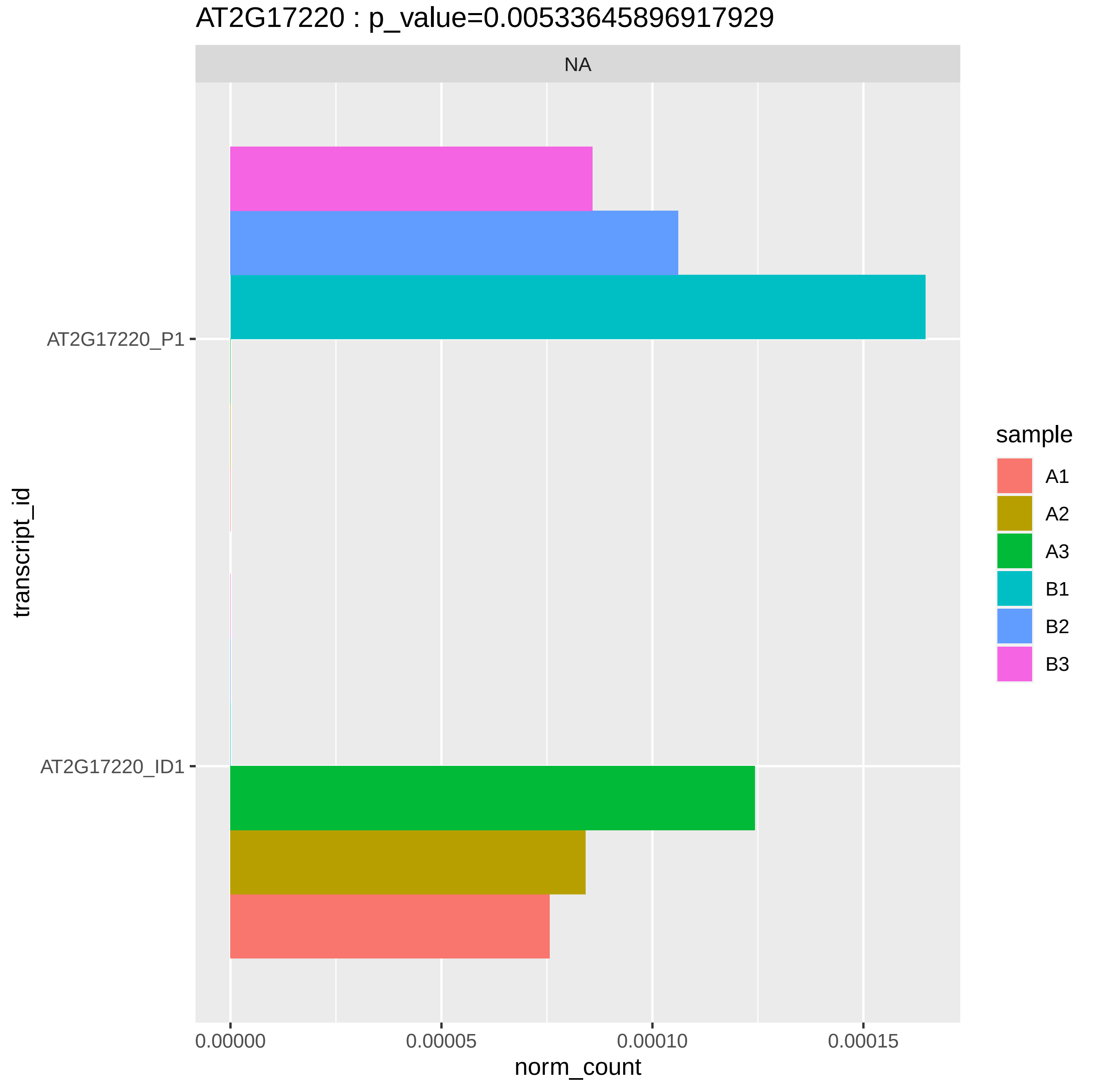

AT2G17220 : p_value=0.00533645896917929
NA
transcript_id
AT2G17220_P1
AT2G17220_ID1
0.00000
0.00005
0.00010
0.00015
norm_count
sample
A1
A2
A3
B1
B2
B3

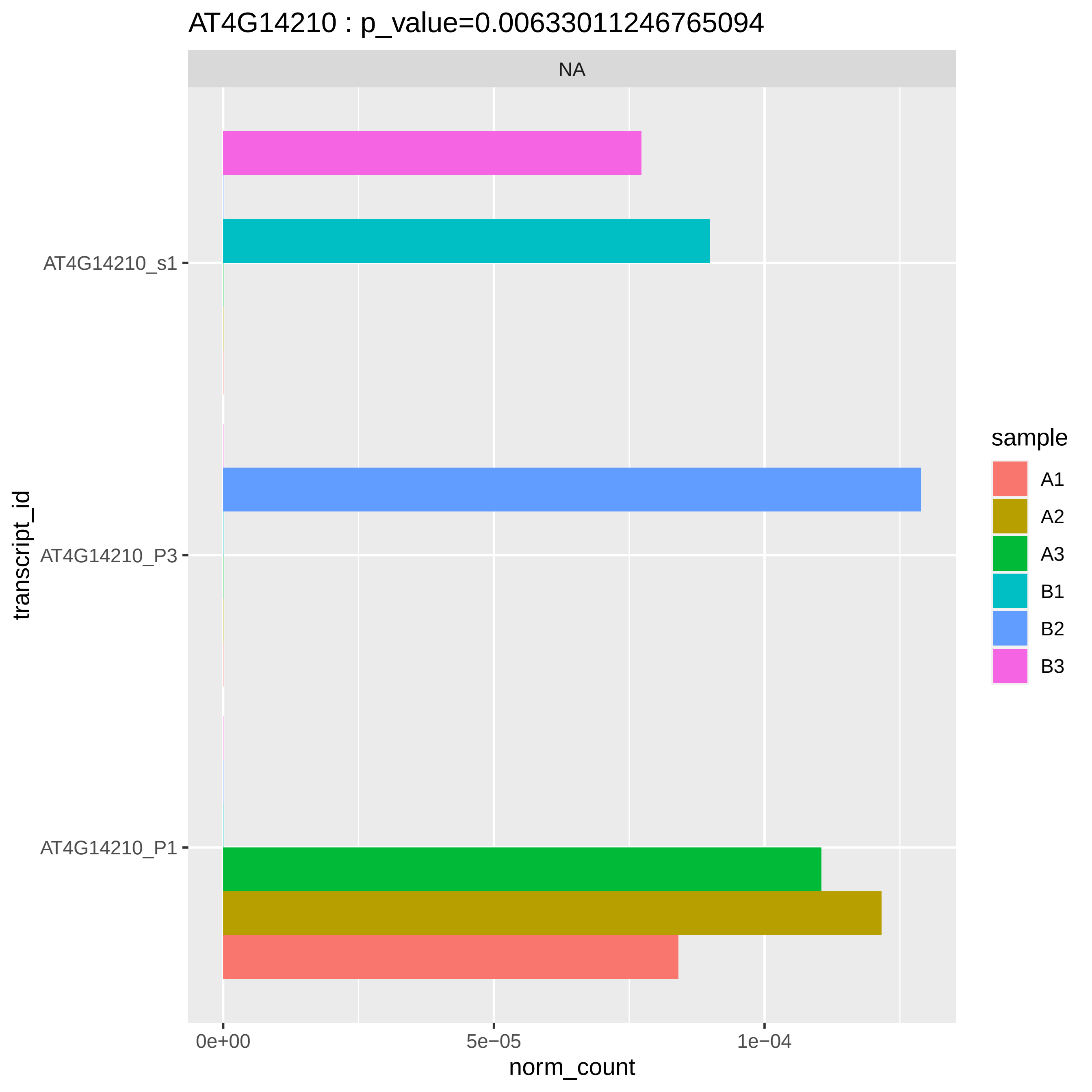

AT4G14210 : p_value=0.00633011246765094
NA
AT4G14210_s1
AT4G14210_P3
AT4G14210_P1
transcript_id
0e+00
5e−05
1e−04
norm_count
sample
A1
A2
A3
B1
B2
B3

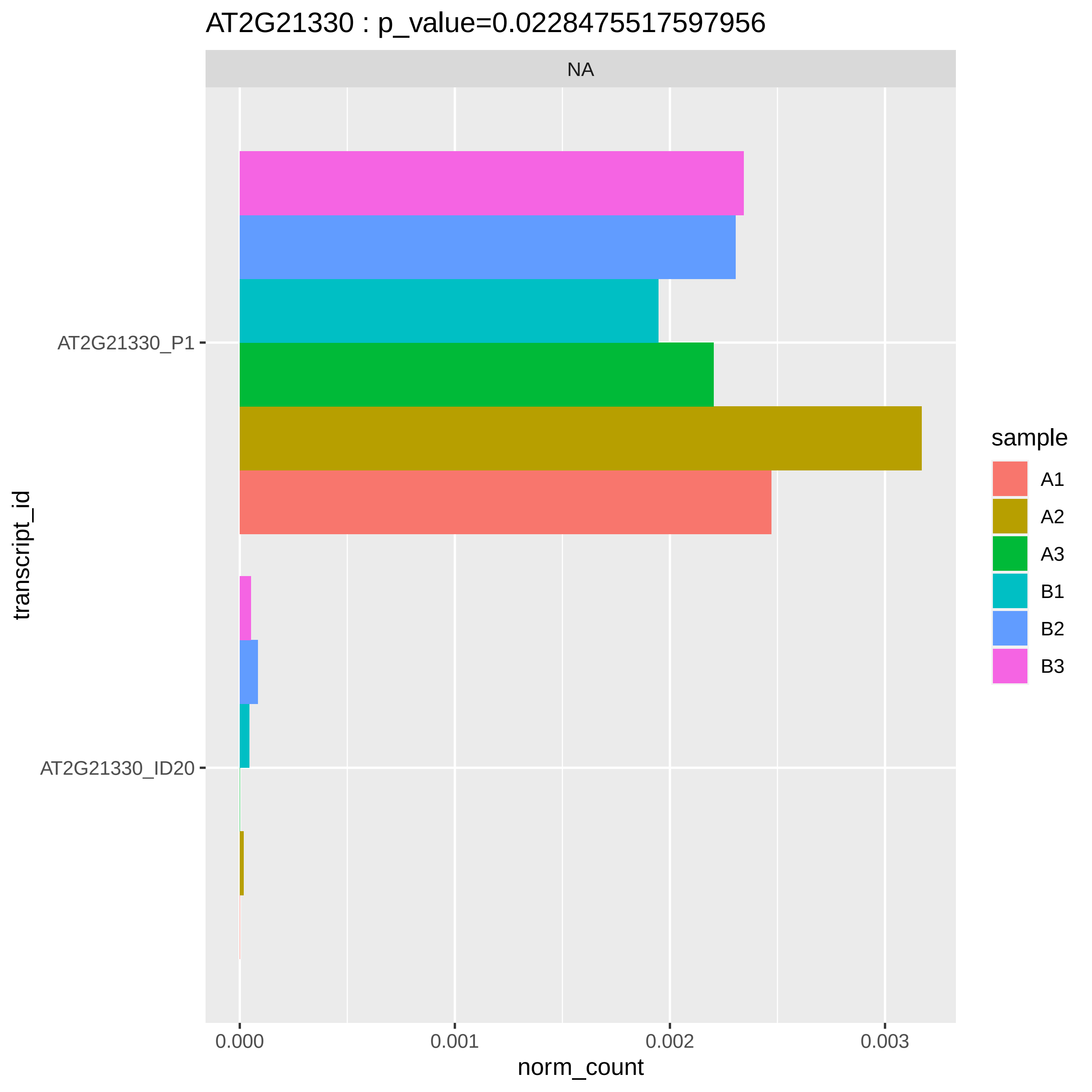

AT2G21330 : p_value=0.0228475517597956
NA
AT2G21330_P1
AT2G21330_ID20
transcript_id
0.000
0.001
0.002
0.003
norm_count
sample
A1
A2
A3
B1
B2
B3

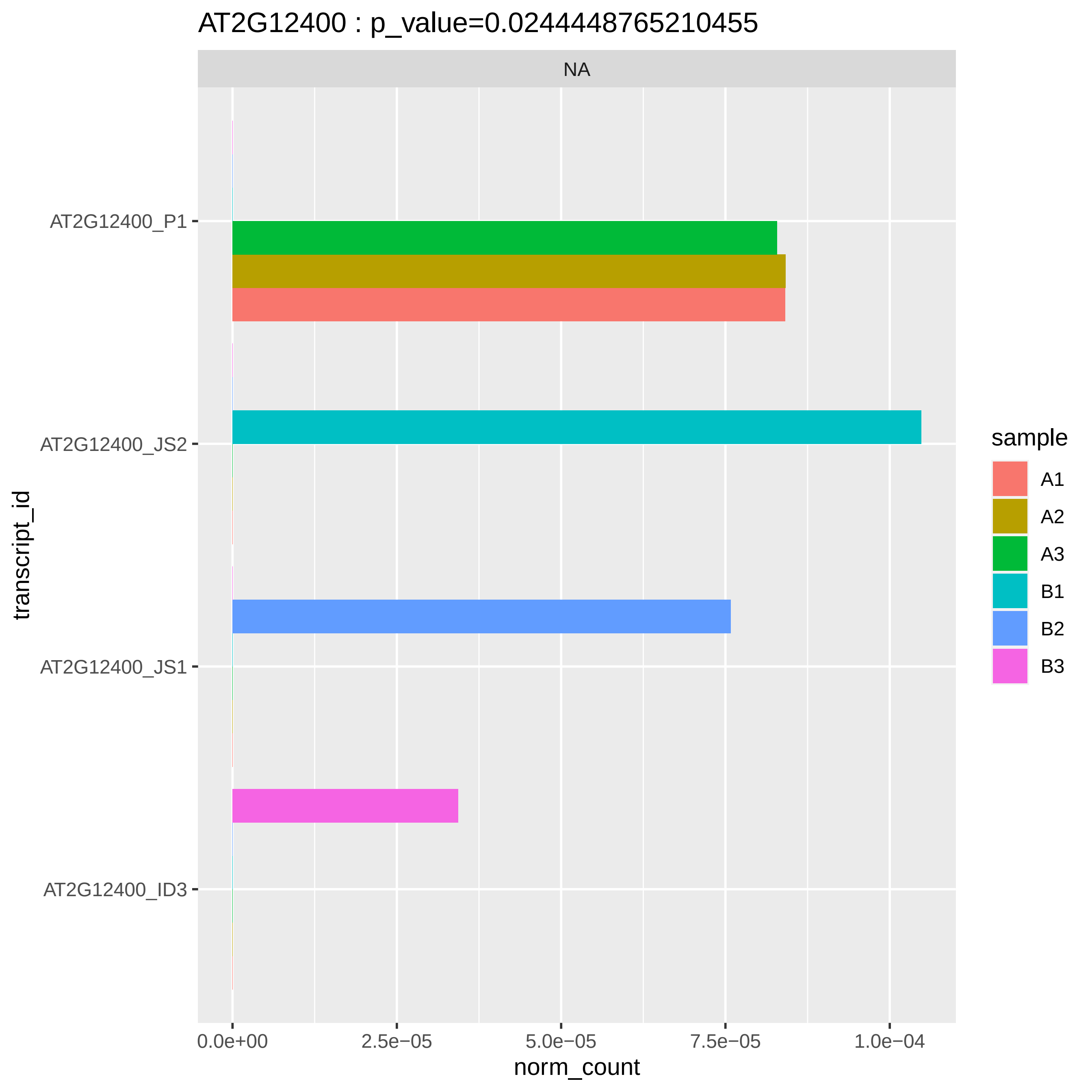

AT2G12400 : p_value=0.0244448765210455
NA
AT2G12400_P1
AT2G12400_JS2
AT2G12400_JS1
AT2G12400_ID3
transcript_id
0.0e+00
2.5e−05
5.0e−05
7.5e−05
1.0e−04
norm_count
sample
A1
A2
A3
B1
B2
B3

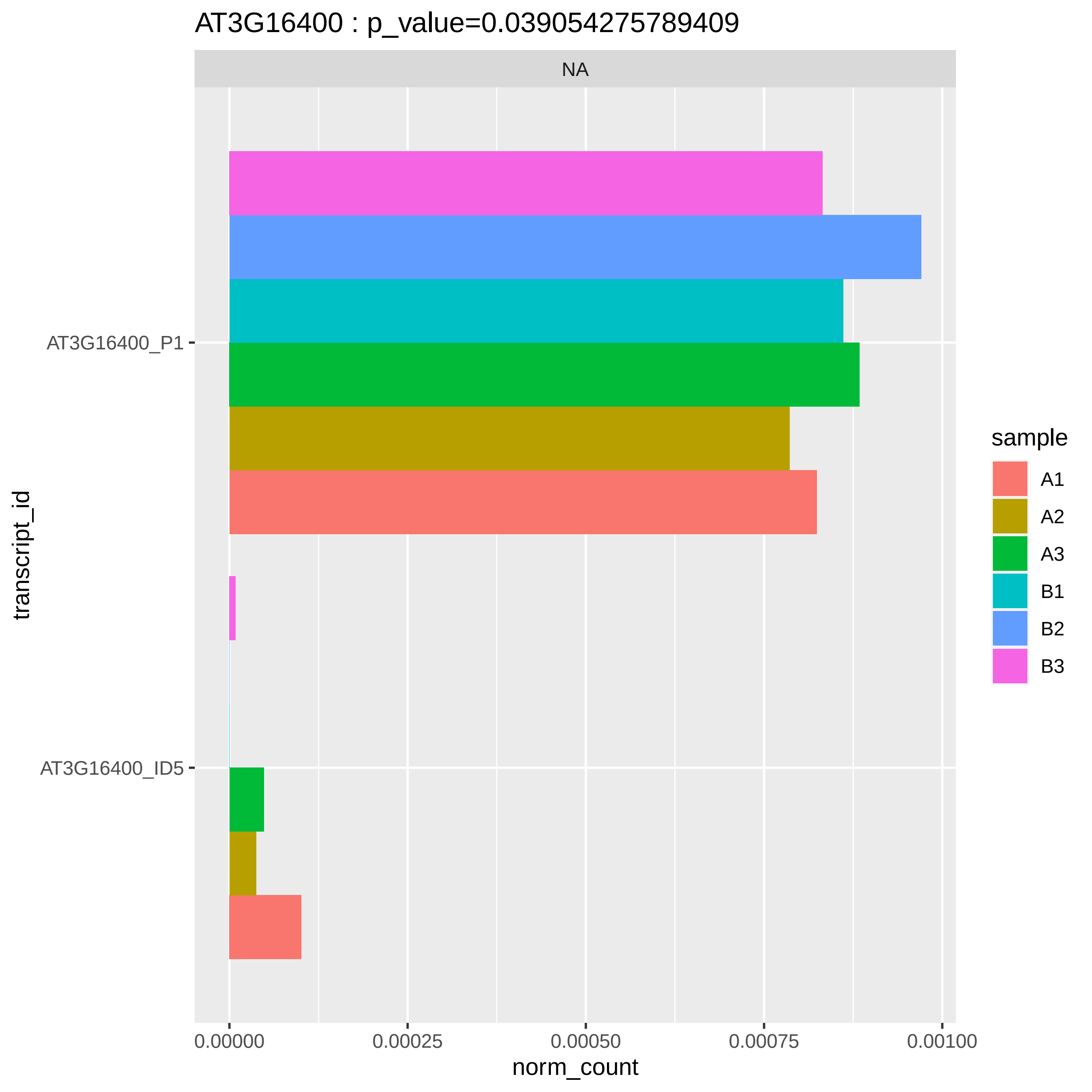

AT3G16400 : p_value=0.039054275789409
NA
transcript_id
AT3G16400_P1
AT3G16400_ID5
0.00000
0.00025
0.00050
0.00075
0.00100
norm_count
sample
A1
A2
A3
B1
B2
B3
