# Supplementary figures and images for: cDNA Transcriptome of Arabidopsis Reveals Various Defense Priming Induced by a Broad-Spectrum Biocontrol Agent Burkholderia sp. SSG
Source: Int J Mol Sci. 2022 Mar 15;23(6):3151. doi: 10.3390/ijms23063151 (PMC8954528; doi:10.3390/ijms23063151)

AT3G51840 : p\_value=0

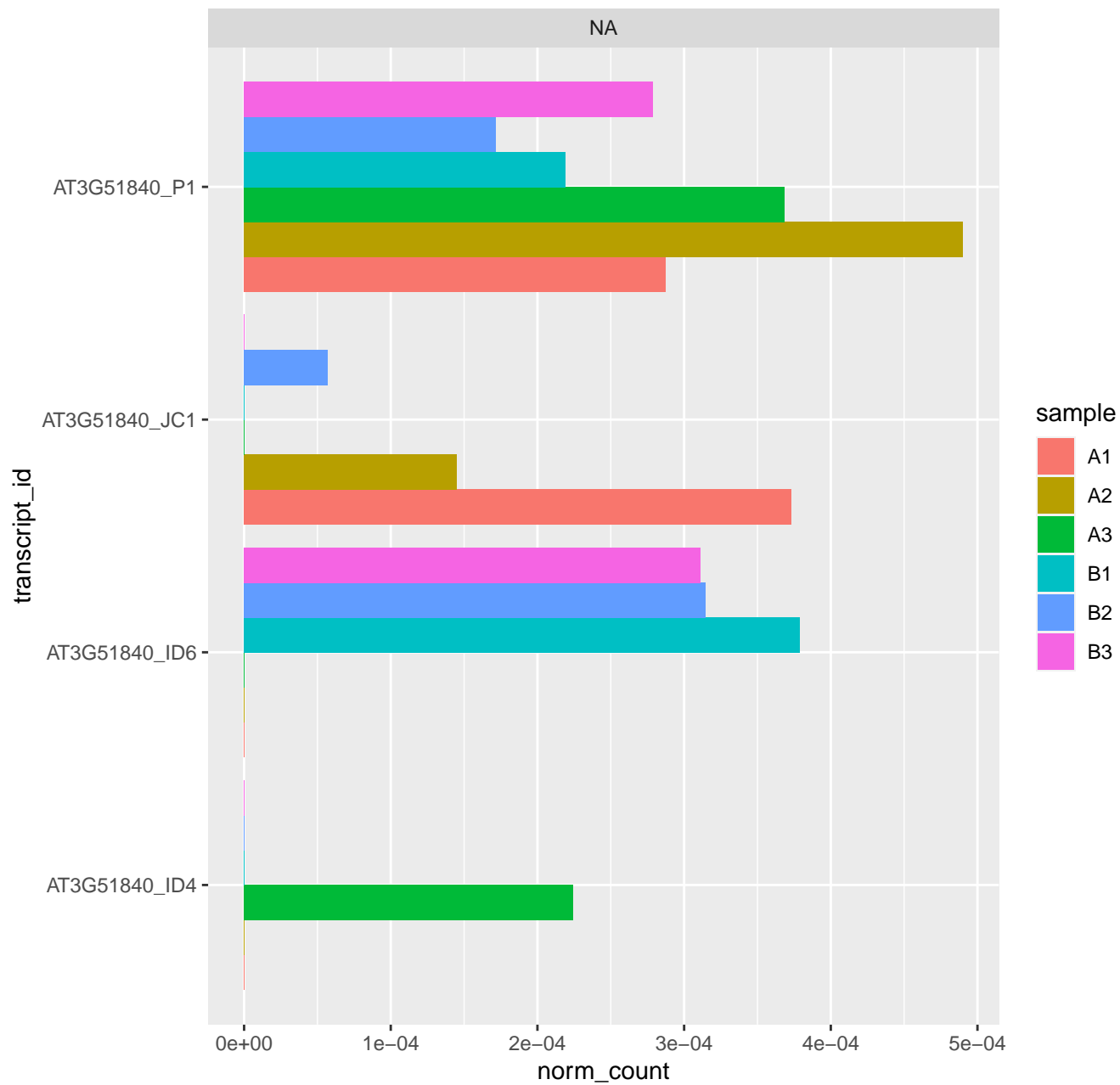

AT4G29010 : p\_value=1.07620672814557e-05

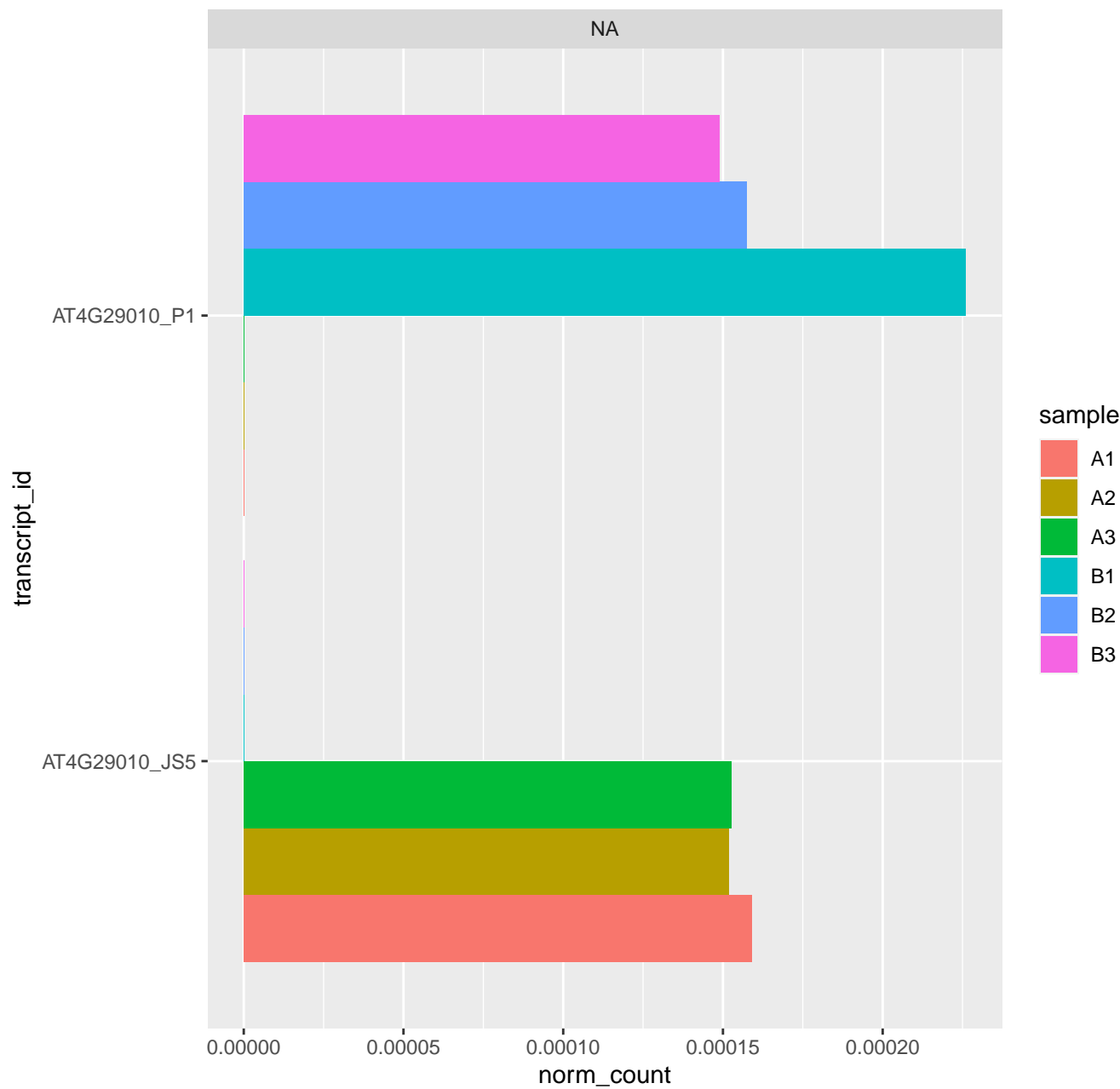

AT4G32410 : p\_value=2.57996591192337e-05

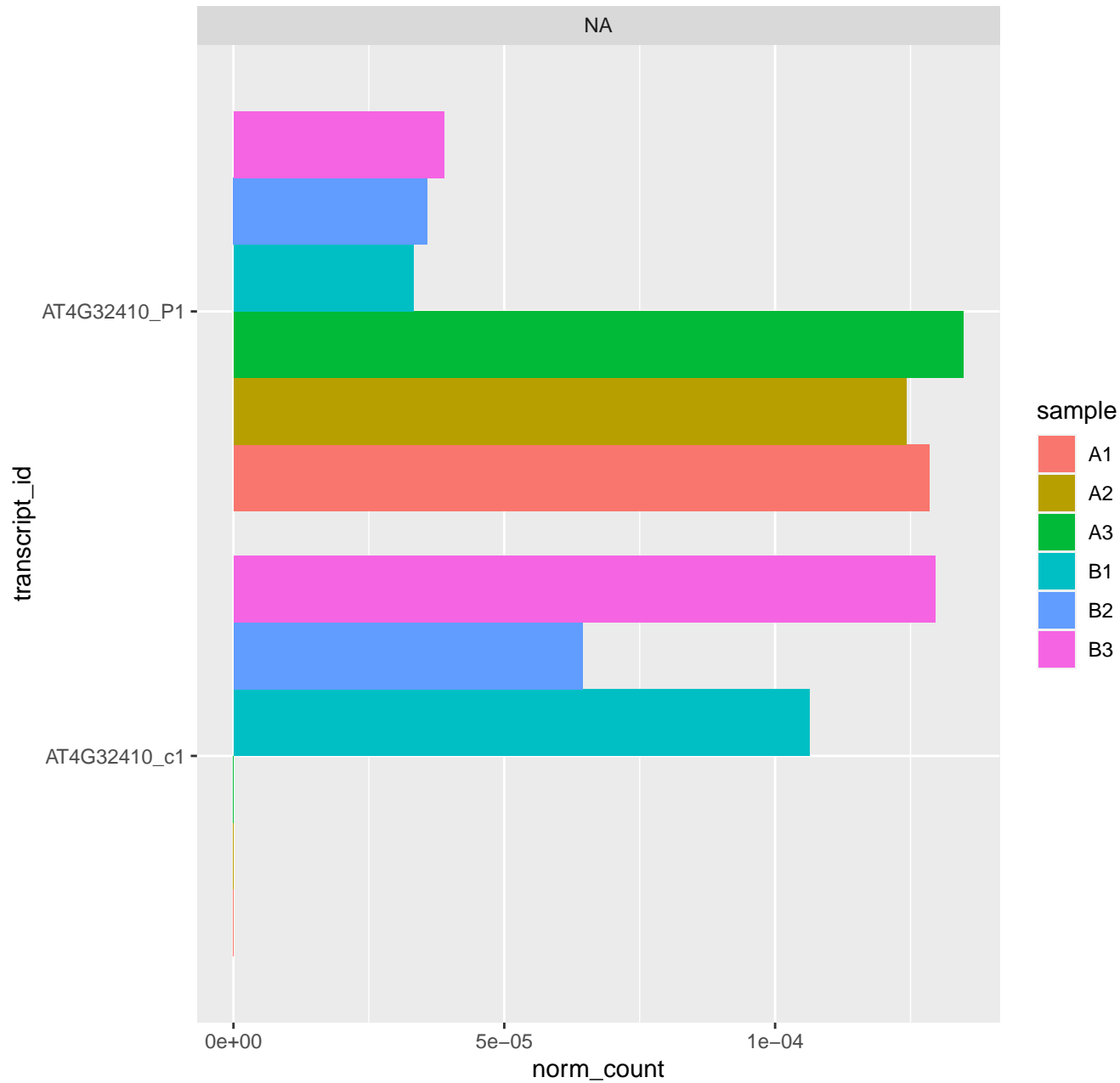

AT5G65780 : p\_value=0.00135859487455278

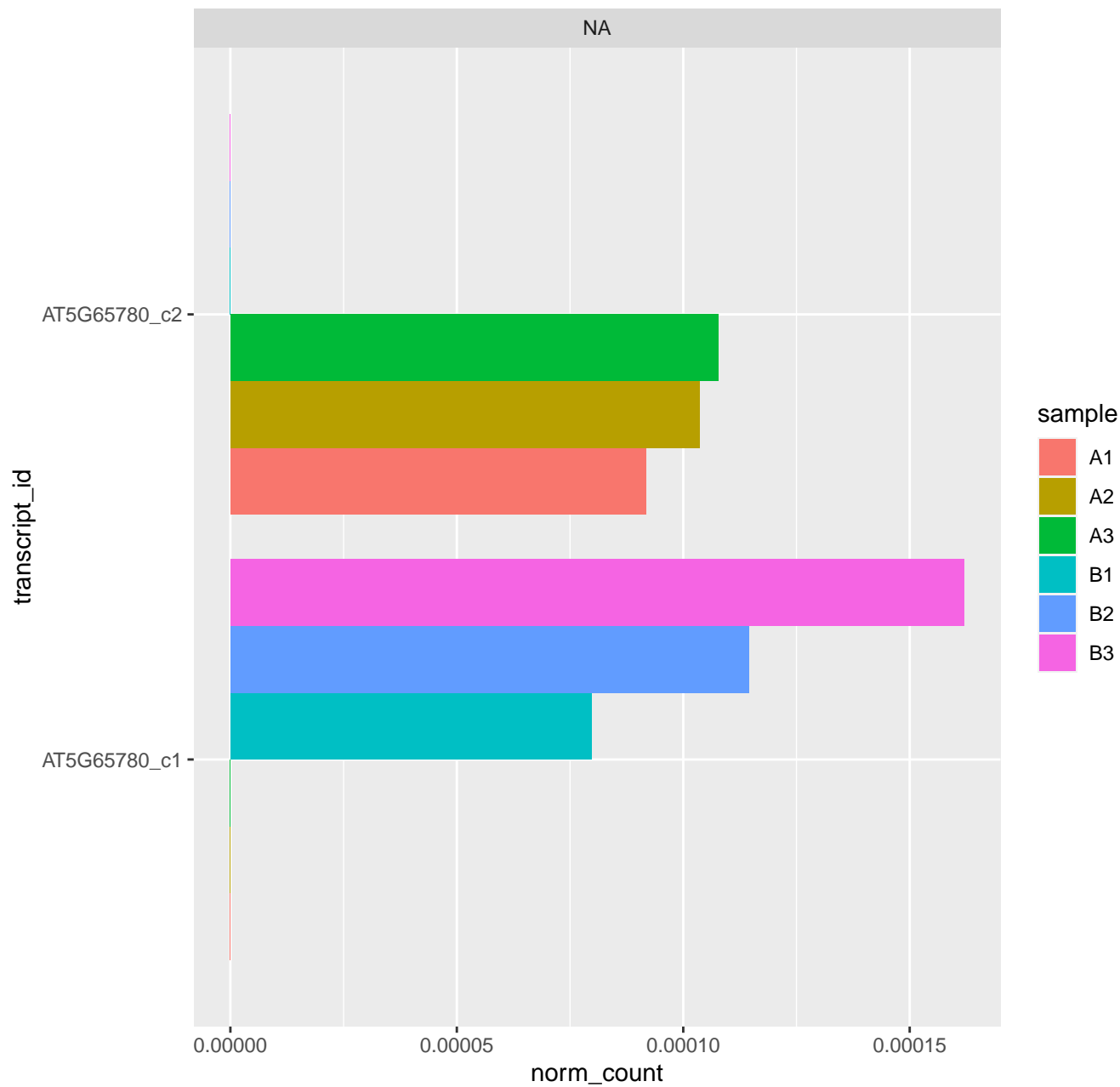

AT5G46020 : p\_value=0.0178428387246834

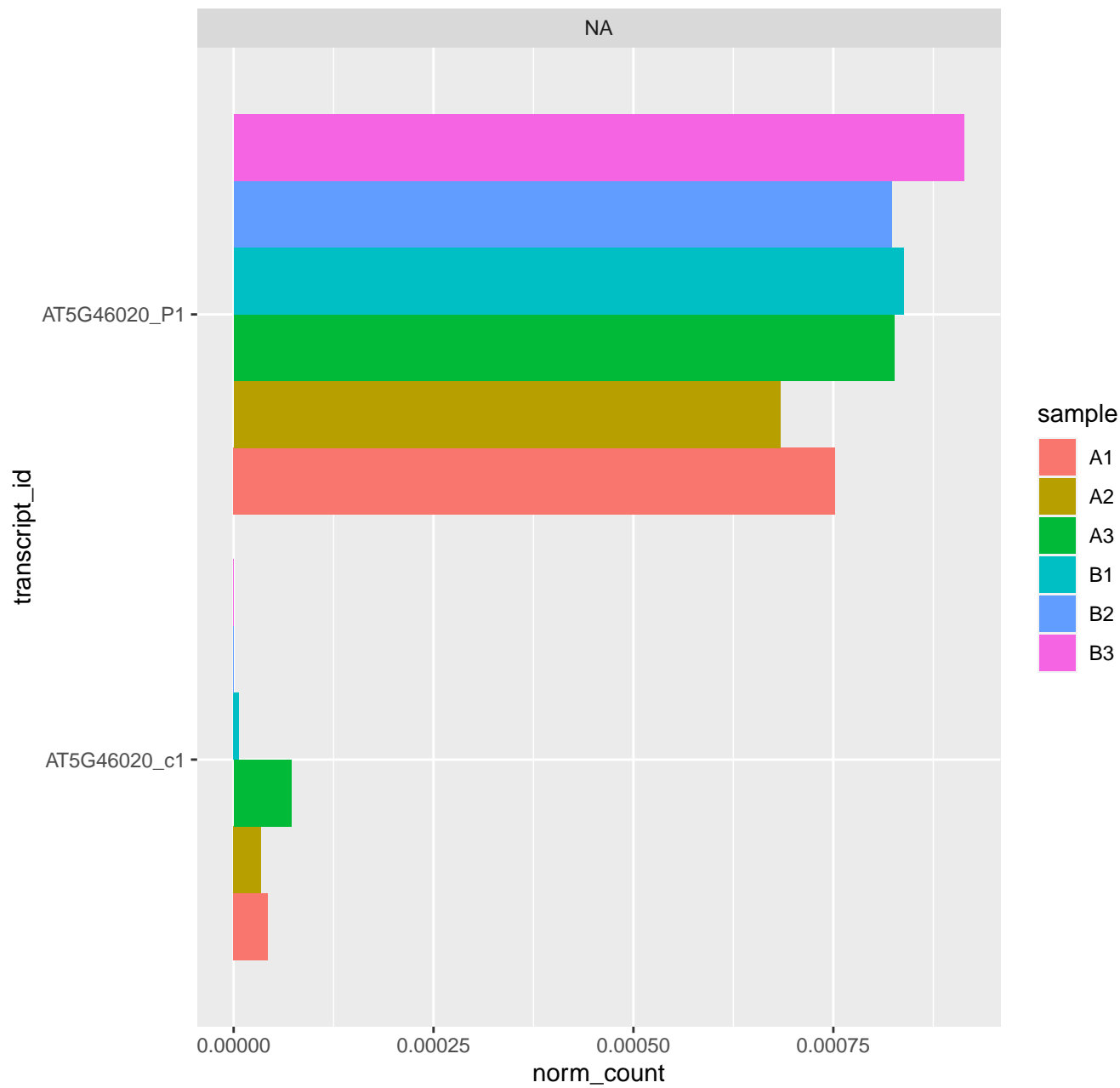

AT4G37990 : p\_value=0.0246413776245916

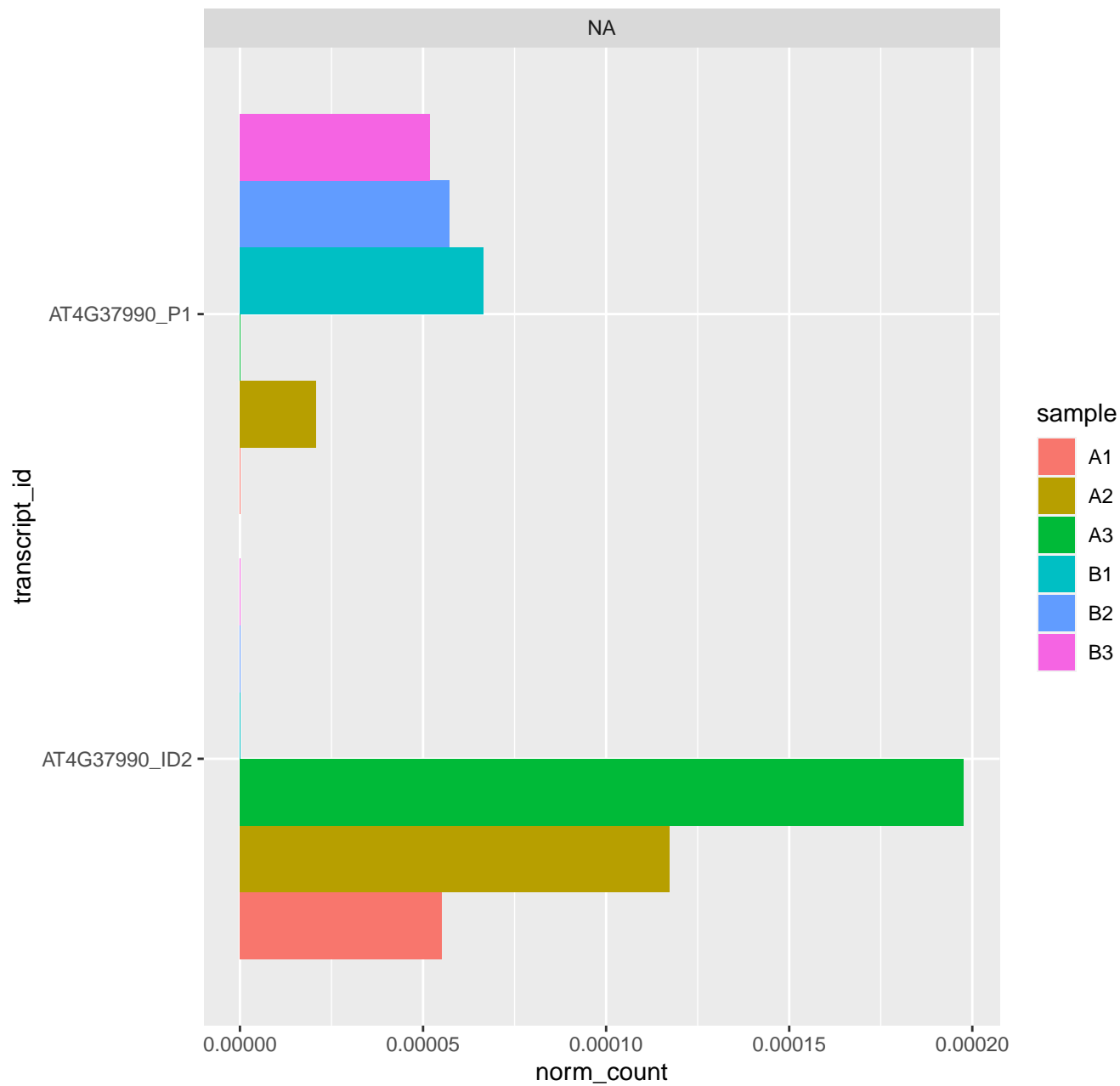

AT1G26440 : p\_value=0.0253219244623181

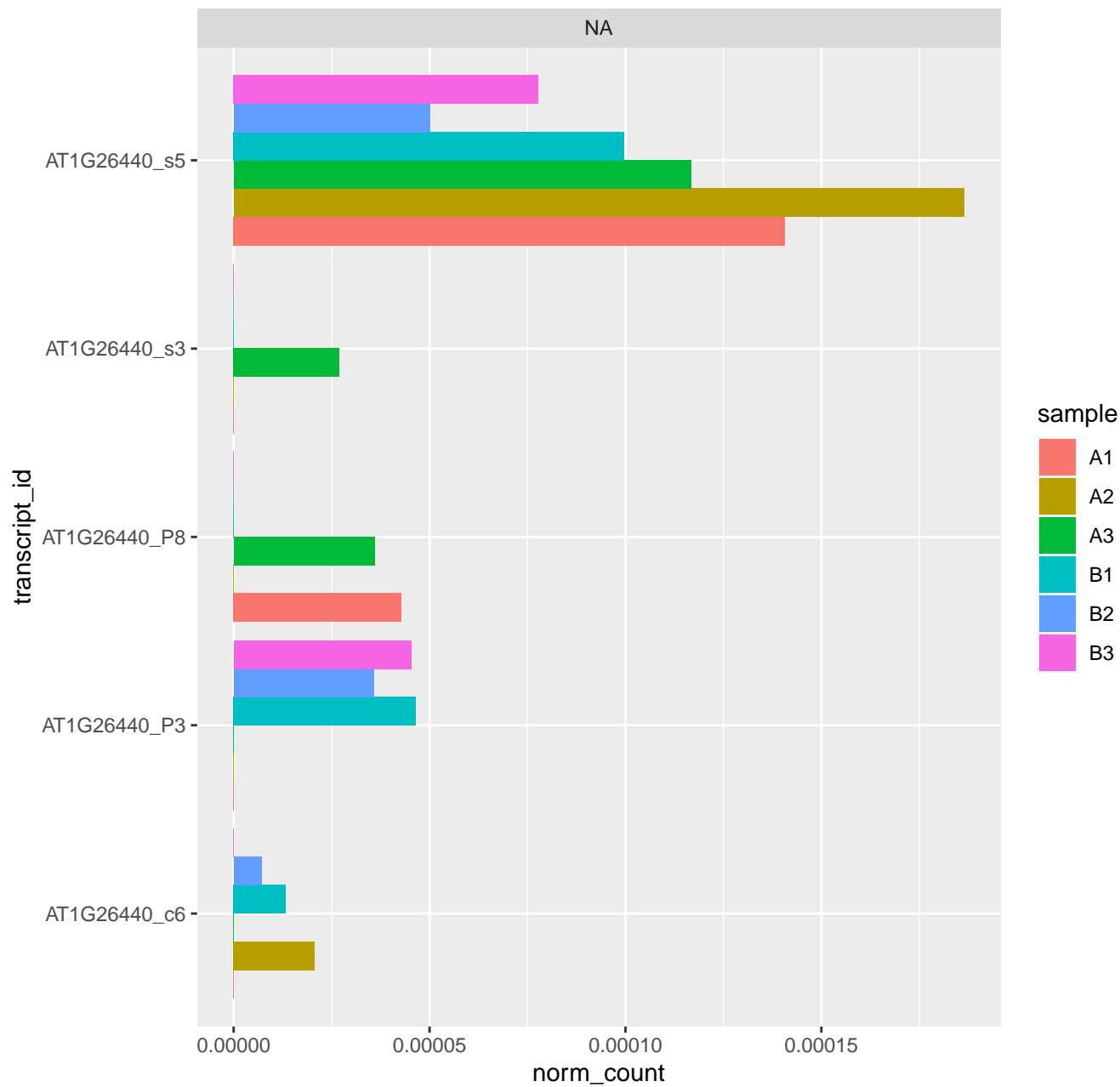

NA

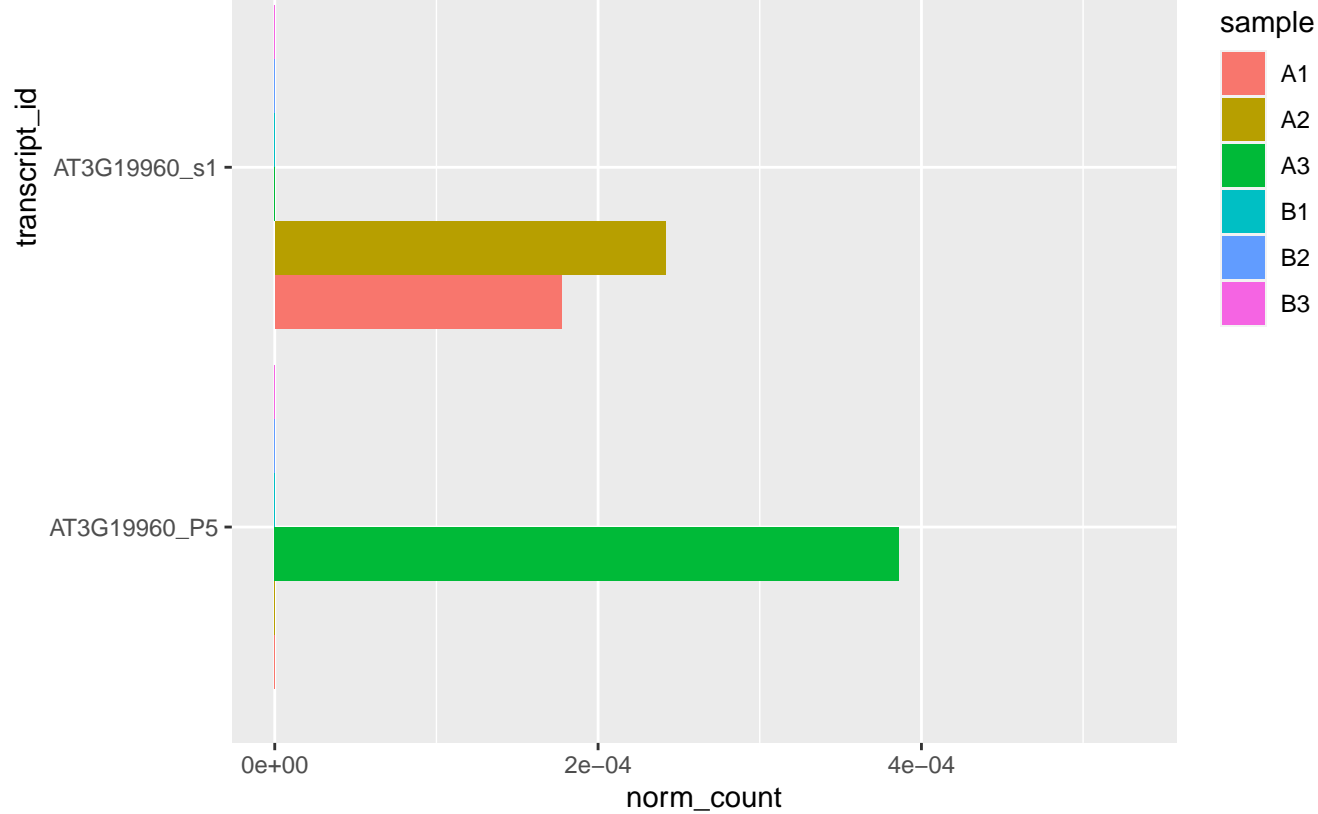

AT2G26910 : p\_value=0.0372221159090733

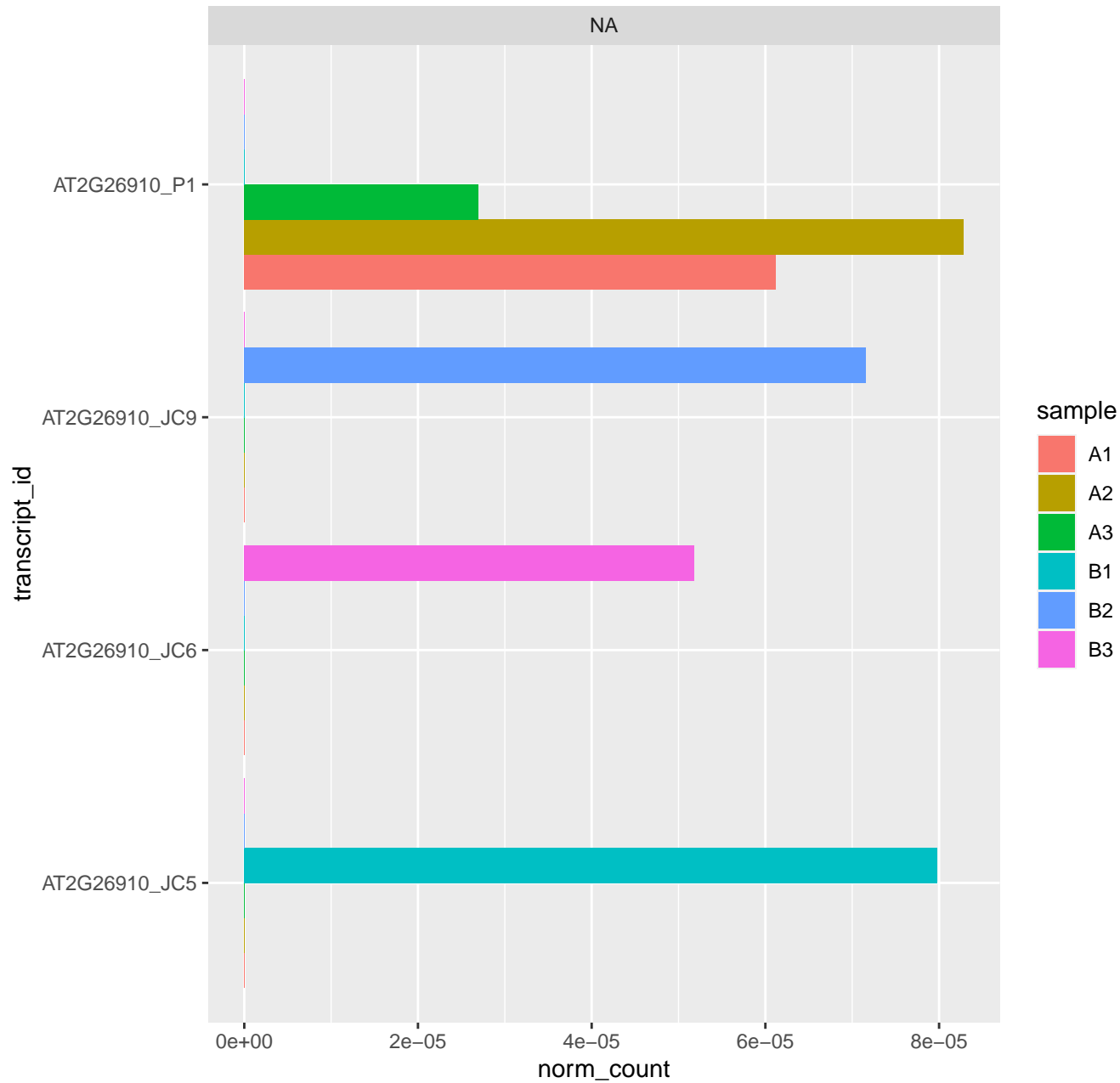

Supplement: Supplementary file 1 [file ijms-23-03151-s001.zip › Sumplementary/Fig. S7.pdf]
